# Theory on the Mechanism of DNA Renaturation: Stochastic Nucleation and Zipping

**DOI:** 10.1371/journal.pone.0153172

**Published:** 2016-04-13

**Authors:** Gnanapragasam Niranjani, Rajamanickam Murugan

**Affiliations:** Department of Biotechnology, Indian Institute of Technology Madras, Chennai, India; Hong Kong University of Science and Technology, HONG KONG

## Abstract

Renaturation of the complementary single strands of DNA is one of the important processes that requires better understanding in the view of molecular biology and biological physics. Here we develop a stochastic dynamical model on the DNA renaturation. According to our model there are at least three steps in the renaturation process viz. nonspecific-contact formation, correct-contact formation and nucleation, and zipping. Most of the earlier two-state models combined nucleation with nonspecific-contact formation step. In our model we suggest that it is considerably meaningful when we combine the nucleation with the zipping since nucleation is the initial step of zipping and nucleated and zipping molecules are indistinguishable. Nonspecific contact formation step is a pure three-dimensional diffusion controlled collision process. Whereas nucleation involves several rounds of one-dimensional slithering and internal displacement dynamics of one single strand of DNA on the other complementary strand in the process of searching for the correct-contact and then initiate nucleation. Upon nucleation, the stochastic zipping follows to generate a fully renatured double stranded DNA. It seems that the square-root dependency of the overall renaturation rate constant on the length of reacting single strands originates mainly from the geometric constraints in the diffusion controlled nonspecific-contact formation step. Further the inverse scaling of the renaturation rate on the viscosity of reaction medium also originates from nonspecific contact formation step. On the other hand the inverse scaling of the renaturation rate with the sequence complexity originates from the stochastic zipping which involves several rounds of crossing over the free-energy barrier at microscopic levels. When the sequence of renaturing single strands of DNA is repetitive with less complexity then the cooperative effects will not be noticeable since the parallel zipping will be a dominant enhancing factor. However for DNA strands with high sequence complexity and length one needs to consider the underlying cooperative effects both at microscopic and macroscopic levels to explain various scaling behaviours of the overall renaturation rate.

## Introduction

The biological function of DNA depends largely on its double stranded helical structure and its ability to unwind and rewind in a reversible manner. The double stranded structure of DNA (dsDNA) is mainly stabilized by weak hydrogen bonds between the nitrogen bases of complementary single strands (c-ssDNAs) and the hydrophobic forces arising from the base-stacking within the core of dsDNA polymer [1–2] These weak interactions melt down upon heating the solution containing dsDNA beyond the melting temperature which in turn yields the corresponding c-ssDNAs. These single strands exactly reunite (hybridize) back into their original double stranded helical form upon cooling the solution below the melting temperature. Melting temperature of dsDNA is defined as the temperature at which precisely half of the dsDNA melts into corresponding c-ssDNAs. Several molecular biological processes such as transcription, translation and replication and *in vitro* laboratory techniques are solely based on the reversible unwinding-rewinding property of dsDNA. Understanding the dynamics and mechanism of renaturation of c-ssDNAs in solution is important in recombination, design of primers for polymerase chain reaction, design of oligonucleotide probes for microarray chips, various membrane blotting techniques and other related DNA fingerprinting technologies [2–5]. In this context, several models describing the process of renaturation of c-ssDNAs in aqueous solution have been developed and experimentally verified [6–29]. Detailed understanding of the mechanism of renaturation of c-ssDNA at microscopic level is one of the important contemporary topics of interest in molecular biology and biological physics.

**Fig 1:**
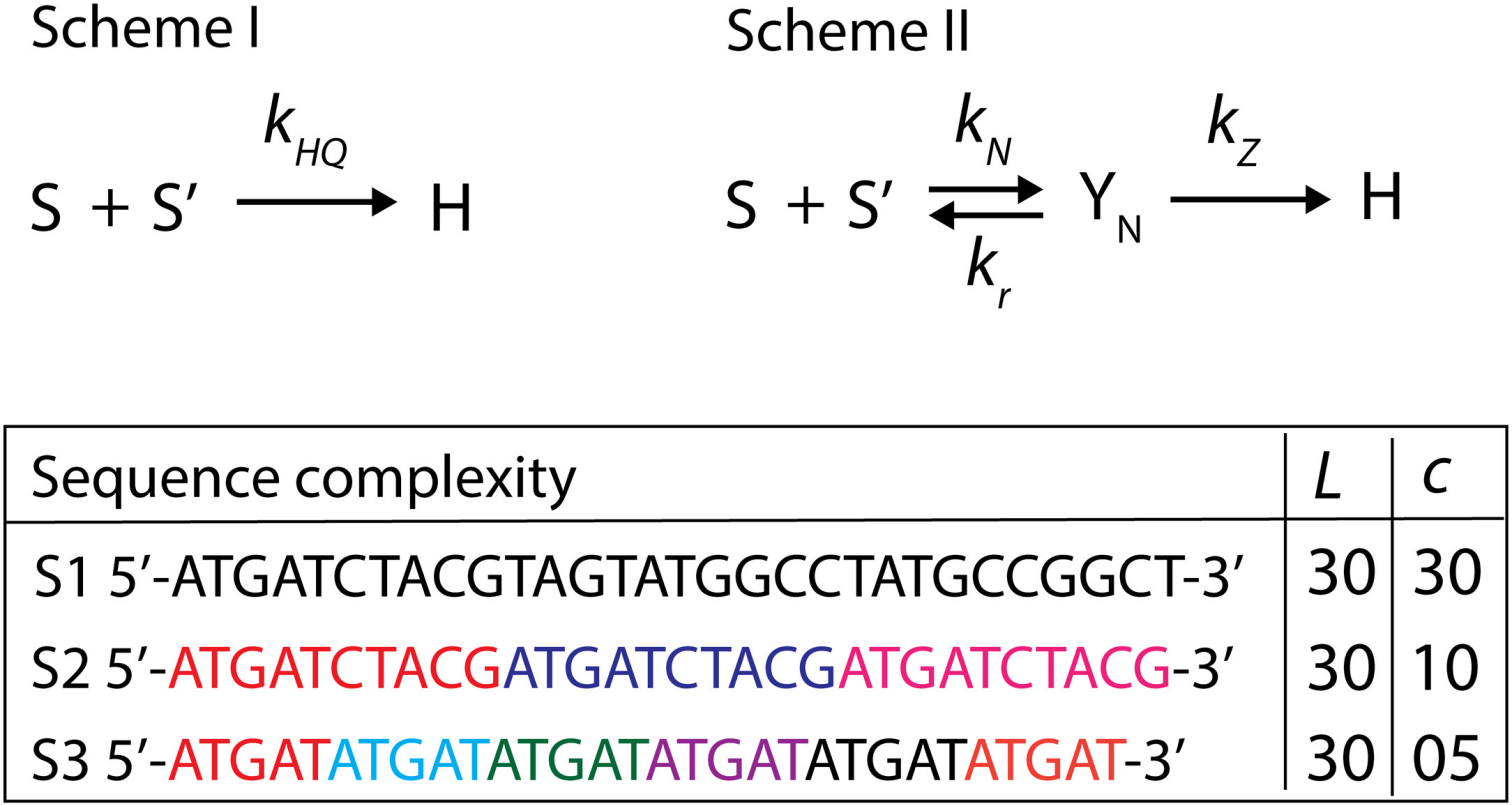
Earlier models on DNA renaturation kinetics. Renaturation of c-ssDNA strands was initially modelled as one-step bimolecular collision rate process as in **Scheme I** with an overall bimolecular association rate of *k*_*HQ*_. *S* and *S’* are the concentrations of c-ssDNA and *H* is the concentration of completely renatured dsDNA. According to this model **Scheme I** the overall renaturation rate *k*_*HQ*_ should scale with the length of the reacting c-ssDNA strands in a linear manner. However experiments revealed a square-root dependency of the renaturation rate on the length of reacting c-ssDNA strands. To comply with the experimental observation a two-step mechanism was proposed as in **Scheme II** which comprised of nucleation and zipping. In this mechanism the nucleation rate (*k*_*N*_) is inversely proportional to the square-root of the length of c-ssDNA strands. This scaling seems to emerge as a consequence of excluded volume effects of c-ssDNA polymer. Whereas the zipping rate (*k*_*Z*_) is directly proportional to the length of c-ssDNA strands (*L*) and inversely proportional to the sequence complexity (*c*). Since the overall renaturation rate is directly proportional to both *k*_*N*_ and *k*_*Z*_ one observes a square-root dependency of the overall renaturation rate on the length of c-ssDNA strands. To generalize nucleation is modelled as a reversible process with a dissociation rate constant *k*_*r*_. Here *Y*_*N*_ is the concentration of the nucleus. **Sequence complexity** of c-ssDNA is defined as the length of DNA with unique sequence pattern. For example consider sequences S1, S2 and S3 all with length of *L* = 30 bases. By definition the sequence complexity of S1 is *c* = 30 bases. Complexity of S2 is *c* = 10 bases since it has 3 repeats of ATGATCTACG with 10 bases length. In the same way, the complexity of S3 is *c* = 5 bases since it has 6 repeats of ATGAT with 5 bases length. The copy numbers *ρ* = *L*/*c* of S1, S2 and S3 are 1, 3 and 6 respectively. The zipping rate in two-step renaturation models as in **Scheme II** is directly proportional to this copy number *ρ*. This means that the overall renaturation rate is inversely proportional to the sequence complexity of the reacting c-ssDNA strands.

Renaturation of c-ssDNAs was initially thought [6] as one-step bimolecular second order chemical kinetic process as in **Scheme I** of Fig 1. Several experimental observations could not be explained by a simple one-step second order kinetics. One of such observations is that irrespective of the experimental conditions the overall bimolecular rate constant was directly proportional to the square-root of the average length of c-ssDNAs and inversely proportional to its sequence complexity. Whereas a one-step process predicted that the bimolecular collision rate was directly proportional to the average length of c-ssDNA. To comply with various experimental observations, Wetmur and Davidson [6] suggested a detailed two-step renaturation model that comprised of nucleation and zipping steps as in **Scheme II** of Fig 1. They had shown that the overall second order rate constant associated with the renaturation phenomenon could be expressed as a function of the average length of the sheared DNA and sequence complexity of the reacting c-ssDNAs. Here the sequence complexity is defined as the length of DNA with unique nucleotide sequence pattern [Fig 1].

Detailed renaturation studies on the sheared genomic DNA suggests [2] the presence of two different types of genomic sequences viz. nonrepetitive and repetitive. Here the degree of repetitiveness can vary from moderate to high. Moderately repetitive DNA contains short sequences that are repeated 10−10^3^ times whereas highly repetitive part of the genomic DNA contains several thousand repeats of short sequences with length of <100 bases (1 base = 3.4 x 10^-10^m). The overall length of nonrepetitive sequences (the sequence complexity) increases along with the genome size and it seems to attain a plateau at ~2 x 10^9^ bases [2]. Wetmur and Davidson further formulated a theoretical model on the renaturation phenomenon according to which the overall bimolecular rate was directly proportional to the nucleation rate apart from the ratio between the average length of c-ssDNA and its sequence complexity. They argued that the nucleation rate must be inversely proportional to the square-root of the average length of c-ssDNA. As a consequence of these facts, the experimentally observed overall bimolecular rate associated with renaturation is directly proportional to the square-root of the length of c-ssDNA. They further suggested that the inverse scaling of the nucleation rate on the length of c-ssDNA polymer must be owing to either the thermodynamic excluded volume effects associated with the intra-strand dynamics or stearic hindrance associated with the diffusion controlled interpenetration of c-ssDNAs that is essential for the nucleation step.

Here one should note that the two-step model of Wetmur-Davidson will be inconsistent whenever the sequence complexity has same magnitude as that of the length of c-ssDNA. Subsequent experimental studies on the renaturation phenomenon were mainly focussed [16–28] on unravelling the molecular mechanisms and the underlying thermodynamics and kinetics aspects. In line with these experiments, several theoretical and computational models [6, 7, 14–17, 23–27] were also developed to explain the observed scaling behaviours of the overall second order renaturation rate on the size of reacting c-ssDNAs, temperature, ionic strength and viscosity of the reaction medium. Recent studies [23–27] considered either the excluded volume effects acting on the intrastrand dynamics or stearic hindrance associated with the diffusion controlled interpenetration of c-ssDNAs to explain the observed scaling behaviours of the overall renaturation rate on the length of c-ssDNA strands. Diffusion based models provide correct viscosity dependence of overall renaturation rate constant compared to the models based on the framework of transition state theories (TST).

Recently nucleation step in renaturation was modelled as an escape over free energy barrier [27] within the framework of Kramer’s theory that deals with the dynamics of Brownian particle over a potential energy barrier. It was argued [27] that the square root dependency of nucleation rate on the length of c-ssDNA mainly originates from the entropic component of the free energy barrier associated with the Kramer’s escape problem. Though this approach appeared to be a reasonable one, nature of the reaction coordinate and potential energy barrier associated with the renaturation process were not clearly defined. Moreover the exact connection between the entropic component of the free energy barrier and the observed scaling behaviour was not clearly established in detail. Here the entropic barrier associated with the nucleation step must originate from the freely moving single stranded overhangs of colliding c-ssDNA strands.

**Fig 2:**
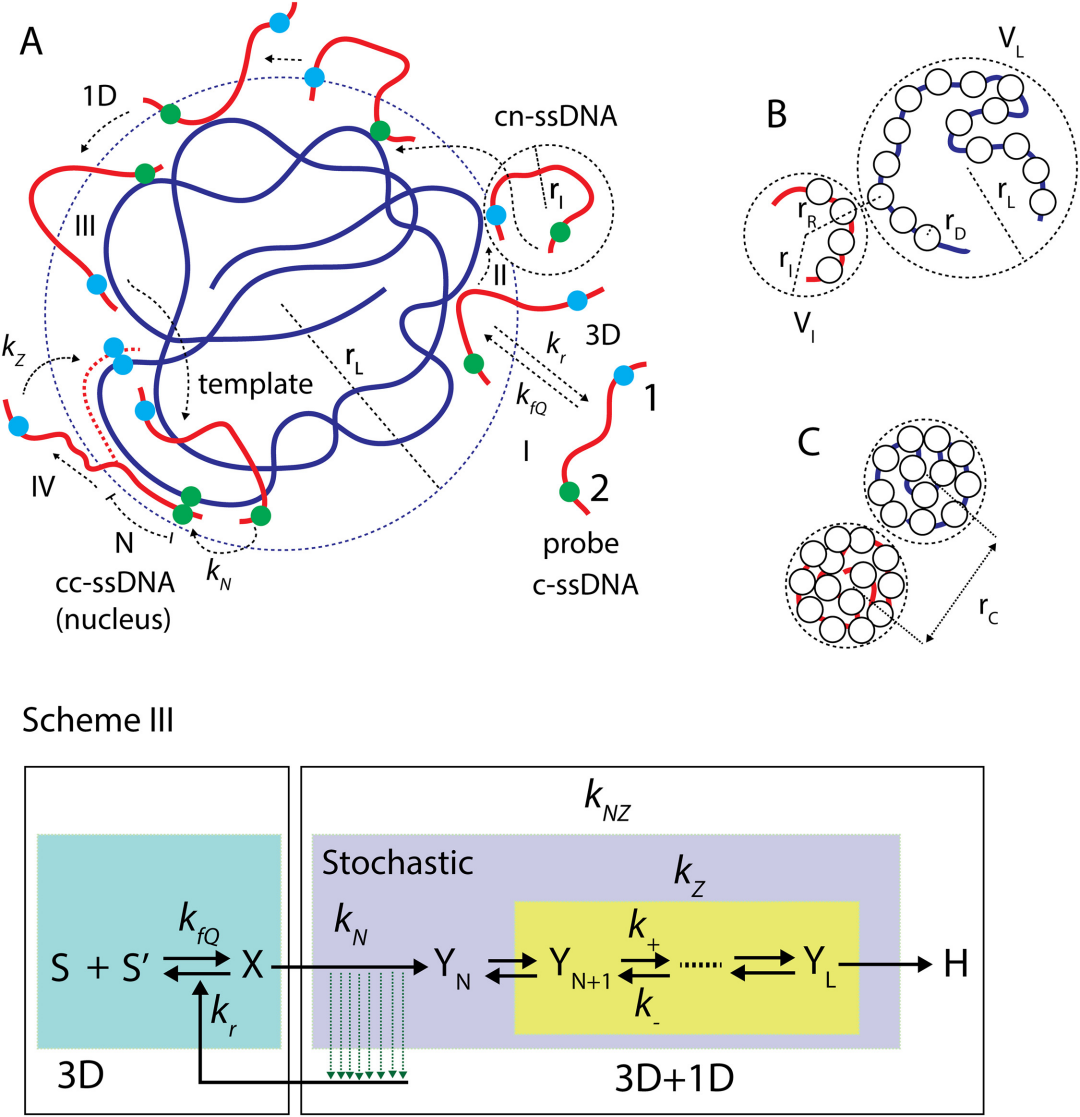
Basic steps of DNA renaturation phenomenon. **A**. Three basic steps in the renaturation of complementary single strands of DNA (c-ssDNA) are viz. nonspecific-contact formation, nucleation and zipping. Two arbitrary locations on the probe c-ssDNA are marked as 1 and 2 (blue and green dots respectively). Nonspecific-contact formation (cn-ssDNA) is purely a three dimensional (3D) diffusion controlled collision rate process (I) where the rate constant associated with the formation of nonspecific-contact scales with the length of colliding c-ssDNA molecules in a square root manner and it scales with the solvent viscosity in an inverse manner. Nucleation involves a one dimensional (1D) slithering dynamics (II) of one strand on the other strand of cn-ssDNA in the process of searching for correct-contact (cc-ssDNA). Internal displacement dynamics through inchworm movements (III) of one complementary strand on the other can facilitate the 1D diffusion dynamics. Upon finding the correct-contact and forming the nucleus, zipping of cc-ssDNA step (IV) follows. **Scheme III.** According to this scheme both the nucleation and zipping are coupled stochastic dynamical processes. In this scheme there are three distinct steps in the process of renaturation viz. 3D diffusion mediated nonspecific contact formation with an on rate of *k*_*fQ*_ and off-rate of *k*_*r*_, 1D and 3D diffusion mediated nucleation step with rate *k*_*N*_ and zipping which is a 1D diffusion process with a rate of *k*_*Z*_. Before forming a successful nucleus with a critical size of *N* bases the colliding c-ssDNA strands undergo several rounds of nonspecific contact formation to form cn-ssDNA, 1D diffusion of one of the cn-ssDNA strands over the other and then dissociation. Upon formation of the nucleus (cc-ssDNA with *N* numbers of correct contacts) zipping process commences. Since nucleated cc-ssDNA is indistinguishable from zipping one, it is more appropriate to combine the nucleation with the zipping with an overall rate of *k*_*NZ*_ = 1/(1/*k*_*N*_ + 1/*k*_*Z*_) rather than with the nonspecific-contact formation step as in **Scheme II**. Conformational state of the reacting c-ssDNA molecules seems to significantly affect the reaction mechanism and scaling relationships associated with the overall renaturation rate on the size of the system. **B**, **C**. We can model the c-ssDNA chains as clusters of nitrogen bases so that the overall bimolecular rate associated with the formation of nonspecific contacts between spatially distributed base-clusters of c-ssDNAs is proportional of the product of concentrations of the total nitrogen bases in c-ssDNA molecules. The cylindrical surface area *C*_*M*_ ~ *2πr*_*D*_*M* of a c-ssDNA molecule with a radius of *r*_*D*_ bases will be confined within the spherical solvent shell with surface area $V_{M}≃4\pi r_{M}^{2}$ (*M* = *L* for template and *M* = *l* for probe c-ssDNA strands) where *r*_*M*_ is the radius of gyration of the respective c-ssDNA molecule. Under strongly condensed state of c-ssDNA one finds that *V*_*M*_ < *C*_*M*_ (**C**) and when the DNA polymer is in a relaxed state then one find that *V*_*M*_ > *C*_*M*_. At a coarse grained level one can model the bases of c-ssDNA as a chain of spherical beads with radius *r*_*D*_. Under relaxed conformational state all these nitrogen base beads are distributed on the surface of the spherical solvent shell that covers a c-ssDNA molecule (**B)**. Under condensed conformational state of c-ssDNA molecules significant fraction of nitrogen base beads will be inaccessible to the inflowing c-ssDNA molecules since they are buried inside the matrix of condensed c-ssDNA (**C**).

**Fig 3:**
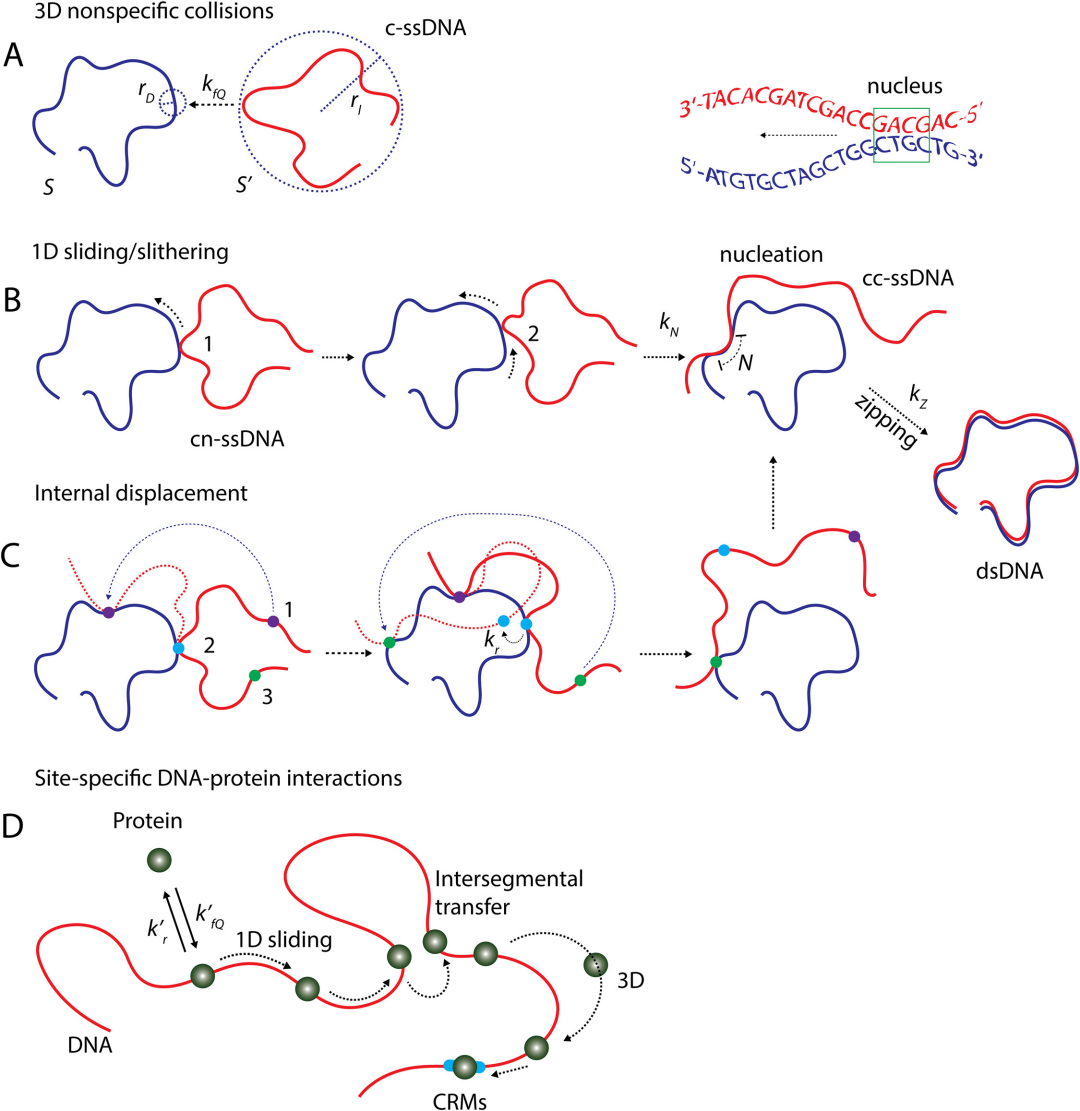
Mechanisms of DNA renaturation. **A**. Collision between c-ssDNA strands leads to the formation of nonspecific contacts (cn-ssDNA) at a diffusion controlled bimolecular collision rate of *k*_*fQ*_ (Q = C for condensed conformation and Q = R for relaxed conformational state of c-ssDNA). **B**, **C**. Slithering and internal displacement mechanism are involved in the nucleation step of renaturation of c-ssDNA strands. Here slithering is a 1D diffusion dynamics (with unit base step size) of one of the cn-ssDNA strands on the other in the process of searching for the correct-contact to initiate nucleation and zipping. Slithering involves local dynamics of individual bases of one strand of cn-ssDNA over the other. Internal displacement mechanism involves inchworm type movement of one of the cn-ssDNA strand over the other. Here two different segments of the same cn-ssDNA strand involved in the inchworm type 1D diffusion dynamics where second nonspecific contact is formed between cn-ssDNA strands before the dissociation the former one with a dissociation rate *k*_*r*_. In the illustration (**C**) three different locations of red colored strand of cn-ssDNA are marked as 1, 2 and 3. Initially position 2 of the probe c-ssDNA strand has a nonspecific contact with the template strand. In this condition the position 3 located on the freely moving overhang of probe strand makes contact with the template strand after which dissociation of the nonspecific contact at position 2 occurs. In this way the probe strand performs an inchworm type movement over the template strand. Occurrence of internal displacements in turn speeds up the 1D diffusion dynamics up to certain extent as in case of the intersegmental transfers via ring closure events associated with the site-specific DNA-protein interactions [31]. Both slithering and internal displacement mechanism are thermally driven stochastic processes which independently contribute to the 1D diffusion coefficient *D*_*o*_. Correct contact formation leads to nucleation with rate *k*_*N*_ beyond the critical nucleus size of *N* ~ 4–7 bases which in turn results in the zipping of cc-ssDNA strands into dsDNA with a rate of *k*_*Z*_. **D**. Slithering seems to be analogous to the sliding mode of searching in the site-specific DNA-protein interactions whereas internal displacement is similar to that of the intersegmental transfer dynamics via ring closure events. Here two distal segments of the same DNA polymer come nearby in 3D space through thermally driven looping dynamics so that the nonspecifically bound protein molecule moves between them. As in DNA renaturation $k_{fQ}^{\prime }$ is the rate constant associated with the forward 3D diffusion mediated nonspecific binding of proteins with DNA and $k_{r}^{\prime }$ is the rate constant associated with the reverse dissociation step. Before reaching the CRMs (specific binding site) the protein molecules perform several rounds of 3D diffusion mediated association with DNA at random locations, 1D diffusion (which includes various modes of facilitating processes such as sliding, hopping and intersegmental transfers) along the DNA polymer and dissociations.

According to the current theoretical understandings over experimental and computational observations [23–29], the renaturation process should have at least three distinct steps namely (**a**) formation of nonspecific contact (**b**) nucleation or correct contact formation and (**c**) zipping [Fig 2A]. In the first step, the reacting c-ssDNAs collide with each other via three-dimensional (3D) diffusion controlled routes. This results in the formation of Watson-Crick (WC) base pairs at random nonspecific contacts between the reacting c-ssDNAs. Such nonspecific WC contacts randomly translocate along c-ssDNAs either via thermally driven one-dimensional (1D) slithering dynamics or internal displacement [29] mechanisms [Figs 2A and 3A–3C] until finding the correct-contact and initiate the nucleation process which is in turn followed by spontaneous zippering of c-ssDNAs.

Here one should note that steps (**a**) and (**b**) are purely stochastic dynamical processes similar to that of the site-specific DNA-protein interactions [17, 30–31]. Additionally zippering step (**c**) will be a stochastic dynamical process since there is a finite probability of dissociation of c-ssDNA strands at each step of the zipping reaction. These mean that one needs to apply stochastic dynamics based arguments rather than merely thermodynamics based ones to explain the observed scaling behaviours and underlying mechanisms. In this paper we will formulate such a stochastic dynamics based theoretical framework of renaturation phenomenon and explain various scaling properties associated with the renaturation rate.

## Results

### Theoretical Formulation of DNA Renaturation Kinetics

At a coarse grained level one can consider the c-ssDNA polymer as a chain of nitrogen base beads with an average bond length of 1 base. The process of renaturation essentially involves collisions between the clusters of nitrogen base beads corresponding to c-ssDNA strands. The basic steps involved in the process of renaturation of c-ssDNA strands viz. (**a**) formation of nonspecific contact (**b**) nucleation or correct contact formation and (**c**) zippering can be well represented by **Scheme III** of Fig 2. Here 3D diffusion controlled collisions between the nitrogen base beads of c-ssDNA strands lead to the formation non-specific contacts between them that results in the formation of cn-ssDNA [Figs 2A and 3A]. Under non-specifically bound condition, the probe strand of cn-ssDNA searches over the template strand for the correct contact via a combination of 1D and 3D diffusion. Here the 1D diffusion comprises of facilitating processes such as slithering and internal displacement dynamics. Slithering dynamics involves [Fig 3B] a random search with unit base step size whereas internal displacement mechanism involves an inchworm type movement of the probe strand over the template strand with a step size of few hundreds to thousand bases [Fig 3C]. One should note that internal displacement mainly depends on the 3D conformational state of the template c-ssDNA strand.

Condensed conformational state of template c-ssDNA always favours internal displacements while relaxed conformational state favours the slithering dynamics. Here slithering and internal displacement mechanism are similar to that of the sliding and intersegmental transfer dynamics as in case of site-specific DNA-protein interactions [Fig 3D]. Intersegmental transfer occurs whenever two distal segments of the same dsDNA chain come close by over 3D space via ring closure events. Under such conditions the non-specifically bound DNA binding protein molecules can move from one segment of DNA to another segment of the same DNA without dissociation. Analogous to intersegmental transfer, internal displacement occurs whenever the overhang parts of the probe strand of cn-ssDNA make contact with some other region of the template strand of cn-ssDNA without dissociation of the previous nonspecific contact. In this way the probe strand of cn-ssDNA can perform an inchworm type movement over the template strand of cn-ssDNA without physical dissociation [Fig 3C]. One should note that the probe strand of cn-ssDNA may dissociate after searching *n* numbers of nonspecific sites on the template strand where *n* is a random variable. These 1D random search processes in combination with several rounds of nonspecific association and dissociation will result in the finding of the correct-contact on the template strand of cn-ssDNA by the probe strand that in turn results in the formation of cc-ssDNA.

Upon formation of cc-ssDNA, the zipping reaction needs to progress against the entropic barrier associated with the single strand overhangs of cc-ssDNA. Nucleation occurs whenever the number of correct contacts in cc-ssDNA exceeds certain critical value (*N*) against the entropic barrier imposed by the freely moving single stranded overhangs [27]. Since the time that is required to locate the correct-contact is a random variable, nucleation rate also will be a random variable. Nucleation will be followed by zipping of cc-ssDNA into a completely renatured dsDNA. Here one should note that both the nucleation and zipping are parts of a continuous process after the formation of cc-ssDNA i.e. there is no clear cut timescale separation or boundary between them. Zipping step will also be a stochastic process since at each step of zipping there is a finite nonzero probability of dissociation of cc-ssDNA. When the c-ssDNA strands are repetitive then the zipping reacting can progress in parallel. As the zipping reaction progresses towards completion, in case of nonrepetitive c-ssDNA strands the stability of cc-ssDNA gradually increases and as a result the probability of dissociation of cc-ssDNA decreases.

In **Scheme III** of Fig 2 [*S*] and [*S’*] are the concentrations (mol/lit, M) of the colliding c-ssDNAs whose lengths are *L* bps (template) and *l* bps (probe) respectively where by definition *L* ≥ *l*, [*X*] is the concentration of c-ssDNAs with nonspecific contacts (cn-ssDNA), [*Y*_*N*_] is the concentration of c-ssDNAs with correct-contact (cc-ssDNA) and nucleation, and [*H*] is the concentration of completely renatured dsDNA form. Here one should note that [*Y*_*N*_] is a special form of [*H*] since the complementary strands are already nucleated and aligned in [*Y*_*N*_]. Since it is very difficult to identify and quantity [*Y*_*N*_] in experiments we consider [*H*] as the main product of renaturation in this paper. Further *k*_*fQ*_ (M^-1^s^-1^) and *k*_*r*_ (s^-1^) are the forward second-order and reverse first-order rate constants associated with the formation of nonspecific contacts between colliding c-ssDNAs, *k*_*N*_ (s^-1^) is the nucleation rate constant and *k*_*Z*_ (s^-1^) is the zippering rate constant. Since [*Y*_*N*_] is a hidden intermediate we consider the overall rate constant associated with both nucleation and subsequent spontaneous zipping as *k*_*NZ*_ which is the inverse of total time required for nucleation and zipping processes together i.e. *k*_*NZ*_ = 1/ (1/*k*_*Z*_ +1/*k*_*N*_). Typical values of the size of nuclei [6] seems to be *N* ~ 4–7 bases. Here we set the subscript *Q* = *C* for condensed conformational state of c-ssDNA and *Q* = *R* for relaxed conformational state of c-ssDNA throughout this paper. The set of differential rate equations associated with **Scheme III** of Fig 2 can be written as follows.

$$d\left[X\right]/dt=k_{fQ}\left[S\right]\left[S\prime \right]-\left(k_{r}+k_{NZ}\right)\left[X\right];\;d\left[H\right]/dt=k_{NZ}\left[X\right];\;d\left[H\right]/dt≃{\bar{k}}_{HQ}\left[S\right]\left[S\prime \right]\;$$ (1)

Since nucleation step involves several rounds of 1D slithering and internal displacement dynamics of one of the cn-ssDNA strand over the other in combination with nonspecific association and dissociation events, *k*_*NZ*_ will be a function of *n*. Here *n* is the number of nonspecific sites scanned by cn-ssDNA strands on each other before dissociation. With this background one can define the overall second order rate constant as ${\bar{k}}_{HQ}=\int_{0}^{L}k_{HQ}p\left(n\right)dn$ where we have defined *k*_*HQ*_ = *k*_*fQ*_*k*_*NZ*_/(*k*_*NZ*_ + *k*_*r*_) and *p*(*n*) is the probability density function associated with the random variable *n*. We will derive the explicit expressions for *k*_*NZ*_ and *p*(*n*) in subsequent sections. Various parameters and variables defined throughout this paper are summarized in Table 1. While deriving the expression for ${\bar{k}}_{HQ}$ we have assumed that the nucleation and zipping are the rate limiting ones so that *d*[*x*]/*dt* ≃ 0 in the timescales of nucleation and zipping.

**Table 1:** Various symbols and their definitions

Symbol	Definition	Remarks
[*S*], [*S’*]	Concentration of complementary single strands of DNA (= [c-ssDNA])	mol/lit, M
[*X*]	Concentration of complementary strands with nonspecific contact between them (= [cn-ssDNA])	M
[*Y*_*N*_]	Concentration of nucleated complementary single strands with nucleus size of *N* base pairs (= [cc-ssDNA])	M
[*H*]	Concentration of double stranded DNA (= [dsDNA]).	M
*k*_*t*_	Smolochowski type 3D diffusion controlled collision rate limit^a^.	M^-1^s^-1^
*D*_*L*_, *D*_*l*_	(= *k*_*B*_*T*/*6πηr*_*M*_) 3D diffusion coefficients associated with c-ssDNA polymers with length *M* bases (*M* = *L*, *l*).	bases^2^s^-1^
*l*_*p*_, *l*_*d*_	Step sizes associated with the 1D slithering (*l*_*p*_ = 1 base) and internal displacement dynamics (*l*_*d*_ > *l*_*p*_)^b^.	bases
*D*_*os*_	(= *l*_*p*_^2^ (*w*_*f*_ *p*_*f*_ + *w*_*r*_ *p*_*r*_)) 1D diffusion coefficient for the searching for the correct contact via slithering ^c^.	bases^2^s^-1^
*D*_*oi*_	(= *l*_*d*_^2^ (*w*_*f*,*d*_ *p*_*f*,*d*_ + *w*_*r*,*d*_ *p*_*r*,*d*_)) 1D diffusion coefficient associated internal displacement^d^.	bases^2^s^-1^
*D*_*o*_	(= *D*_*os*_ + *D*_*oi*_)	bases^2^s^-1^
*r*_*D*_	Radius of DNA cylinder or radius of a nitrogen base bead. (1base = 3.4 x 10^−10^ m)	bases
*L*, *l*	Lengths of template and probe c-ssDNA strands, *L* ≥ *l*.	bases
*r*_*L*_, *r*_*l*_	Radius of gyration of c-ssDNA polymer in aqueous solution whose lengths are *L* (template) and *l* (probe) ^e^.	bases
*δ*_*C*_, *δ*_*R*_	Multiplication factor corresponding to geometric constraints for the collision of c-ssDNA strands ^f^.	dimensionless
*κ*	Onsager radius*.	bases
*ψ*_*Q*_	Multiplication factor for the electrostatic repulsion component associated with the collision of c-ssDNA.	dimensionless
*χ*_*Q*_	Multiplication factor for the overall electrostatic component associated with the collision of c-ssDNA.	dimensionless
*k*_*fQ*_	Bimolecular collision rate constant for the formation of nonspecific contacts (= *k*_*t*_*χ*_*Q*_*δ*_*Q*_), (*Q* = *C*, *R*).	M^-1^s^-1^
*k*_*r*_	Rate constant associated with the dissociation of cn-ssDNA.	s^-1^
*λ*	*(= L/n)* minimum number of 1D slithering of cn-ssDNA of *n* bases required to find the correct contact.	dimensionless
*N*	Critical number of correct contacts in cc-ssDNA that can be considered as nucleus. (*N* ~ 4–7 bases).	bases
*τ*_*N*_	(= *λn*^*2*^*/12D*_*o*_) overall time required for nucleation.	s
*τ*_*Z*_	Zippering time to generate a completely renatured dsDNA from nucleation.	s
*k*_*N*_	Rate constant associated with the formation of nucleus (= 1/*τ*_*N*_, where *τ*_*N*_ is the nucleation time).	s^-1^
*k*_*Z*_	Macroscopic zippering rate constant (= 1/*τ*_*Z*_, where *τ*_*Z*_ is the zippering time).	s^-1^
*k*_*NZ*_	(= 1/(1/*k*_*Z*_ +1/*k*_*N*_) = 1/ (*τ*_*Z*_ + *τ*_*N*_)) coupled stochastic nucleation-zipping rate constant.	s^-1^
*C*_*L*_, *C*_*l*_	Area of cylindrical surface of c-ssDNA (*C*_*L*_ = *2πr*_*D*_^*2*^*L*, *C*_*l*_ *= 2πr*_*D*_^*2*^*l*) whose lengths are *L* and *l* bases.	bases^2^
*V*_*L*_, *V*_*l*_	Area of spherical solvent shell that covers c-ssDNA polymer (*= 4πr*_*M*_^*2*^).	bases^2^
*r*_*C*_, *r*_*R*_	Reaction radius associated with the collision of one c-ssDNA polymer with another c-ssDNA^g^.	bases
*c*	Sequence complexity of c-ssDNA which is the length of unique sequence found in a given stretch of DNA.	bases
*ρ*	(= *L*/*c*) number of repeats in c-ssDNA with length *L* bases and complexity of *c* bases.	dimensionless
*k*_*+*_, *k*_*-*_	Microscopic forward (*k*_*+*_) and reverse (*k*_*-*_) transition rate constants associated with the zipping process.	s^-1^
*K*_*Z*_	(= *k*_*-*_*/k*_*+*_) equilibrium constant associated with the microscopic zipping process.	dimensionless
*D*_*±*_	(= *l*_*p*_^*2*^*k*_*+*_) microscopic diffusion coefficient associated with the zipping process when *k*_*+*_ = *k*_*-*_ where *l*_*p*_ = 1 base.	bases^2^s^-1^
*n*	Number of possible nonspecific contacts scanned by cn-ssDNA before it dissociates into c-ssDNA.	dimensionless
*u*	Number of correct contacts at a given time point in the reaction associated with the zipping cc-ssDNA.	dimensionless
*β*	(= *L*/*l*_*p*_) maximum number of correct contacts in the completely renatured dsDNA.	dimensionless
*k*_*p*_	(= *k*_*+*_*l*_*p*_) microscopic zipping rate constant.	bases s^-1^
*Y*_*A*_	Distance travelled by nonspecific contact via 1D slithering before cn-ssDNA dissociates.	bases
*Y*_*E*_	(= *k*_*p*_*/k*_*r*_) distance travelled in the zipping reaction before cn-ssDNA dissociates.	bases
*k*_*HQ*_	Bimolecular rate constant associated with the complete renaturation ^h^.	M^-1^s^-1^
*L*_*opt*_	optimum value of the length of c-ssDNA at which *k*_*HR*_ will be a maximum ^i^.	bases
*p*(*n*)	Probability density function (pdf) associated with the 1D slithering length (*n*).	pdf
*k*_*sm*_	(= *k*_*t*_*ε*) Smolochowski type 3D collision rate limit associated with nitrogen bases A with T or G with C ^j^.	M^-1^s^-1^
*k*_*2*_	Overall bimolecular renaturation rate in Wetmur-Davidson model ^k^.	M^-1^s^-1^

Notes:

*Onsager radius is defined as the distance between negatively charged phosphate backbones of colliding c-ssDNA chains at which the electrostatic repulsive energy is same as that of the thermal energy.

^a.^ Here colliding molecules are of same in size with no charge on them (= 8*k*_*B*_*T/*3*η* where *k*_*B*_ is the Boltzmann constant, *T* absolute temperature in degree K and *η* is the viscosity of solvent). For T = 298K and *η* ~10^−1^ kgm^-1^s^-1^ one obtains *k*_*t*_ ~ 10^9^ M^-1^s^-1^.

^b.^ Here *l*_*p*_ is the average bond length associated with c-ssDNA polymer and *l*_*d*_ is the average step size associated with the internal displacement mechanism. Here we consider the average step size since the step size associated with the internal displacement is a random variable.

^c.^ Here *w*_*f*_ and *w*_*r*_ are microscopic forwards and reverse transition rates, *p*_*f*_ and *p*_*r*_ are the corresponding transition probabilities and *l*_*p*_ = 1 base is the step size.

^d.^ Here *w*_*f*,*d*_ and *w*_*r*,*d*_ are microscopic forward and reverse transition rates, *p*_*f*,*d*_ and *p*_*r*,*d*_ are the corresponding transition probabilities and *l*_*d*_ > *l*_*p*_ is the average step size.

^e.^ For a linear chain polymer $r_{M}≃\sqrt{Ml_{p}\;/6}$ where *M* = *L*, *l*.

^f.^ (*δ*_*R*_ = *L* (1/*r*_*L*_ + 1/*r*_*l*_)/8, *δ*_*C*_ = (*r*_*L*_ + *r*_*l*_) ^2^/4 *r*_*L*_*r*_*l*._). When *L* = *l*, then one finds that *δ*_*R*_ = *L*/4*r*_*L*_, *δ*_*C*_ = 1.

^g.^ In case of condensed conformational state, *r*_*C*_ = *r*_*L*_ + *r*_*l*_ and in case of relaxed conformational state, *r*_*R*_ = *r*_*D*_ + *r*_*l*_).

^h.^ (= *k*_*fQ*_*k*_*NZ*_/(*k*_*r*_ + *k*_*NZ*_)). Since *k*_*NZ*_ is a function of 1D slithering length (*n*), *k*_*HQ*_also will be a function of *n* and subsequently we find that $k_{HQ}:\to k_{HQ}(n)≃k_{fQ}/(1+nL/Y_{A}^{2}+c/Y_{E})$. Here Q = C, R depending in the conformational state of c-ssDNA strands. ${\bar{k}}_{HQ}:\to \int_{0}^{L}k_{HQ}p(n)dn$. Under relaxed conformational state of c-ssDNA strands when (*pY*_*E*_/*Y*_*A*_) ≪ 1 then as in Eq 15 one obtains ${\bar{k}}_{HR}≃k_{sm}\sqrt{Ll_{p}/c}$ where *k*_*sm*_ = *k*_*t*_*ε*.

^i.^ ($≃Y_{A}^{2}(c+Y_{E})/nY_{E}$) Under relaxed conformational state. Solution of *∂*_*L*_*k*_*HR*_ = 0 for *L*.

^j.^ (Where *L* = *c* = 1). Here we have defined $\varepsilon ≃\chi_{R}(k_{+}/k_{r})\sqrt{3/8}$. Upon comparison with the experimental data **[**6] one finds that *ε*~ 10^−3^.

^k.^ Detailed fitting over the experimental data suggested [6] the empirical form as $k_{2}=3.5\times 10^{5}\sqrt{L/c}$. We denote this by *k*_*HR*_ in our model.

Essentially the first reaction in **Scheme III** of Fig 2 can be thought as collisions between the spatially distributed clusters of nitrogen bases corresponding to the two reacting c-ssDNAs as in case of a mean field approach [27]. As a result, the overall bimolecular rate associated with the formation of nonspecific contacts between spatially distributed base-clusters of c-ssDNAs is proportional of the product of concentrations of the total nitrogen bases in c-ssDNA molecules. The cylindrical surface area *C*_*M*_ ~ *2πr*_*D*_*M* of a c-ssDNA molecule will be confined within the spherical solvent shell with surface area $V_{M}≃4\pi r_{M}^{2}$ (*M* = *L* for template and *M* = *l* for probe) where *r*_*Q*_ is the radius of gyration of the respective c-ssDNA molecule. Under strongly condensed conformational state of c-ssDNA one finds that *V*_*M*_ < *C*_*M*_ and when the DNA polymer is in a relaxed conformational state then one find that *V*_*M*_ > *C*_*M*_. At a coarse grained level one can model the bases of c-ssDNA as a chain of spherical beads with radius *r*_*D*_. Under relaxed conformational state all these nitrogen base beads are distributed on the surface of the spherical solvent shell that covers the entire c-ssDNA molecule [Fig 2B]. Whereas under condensed conformational state of template c-ssDNA significant fraction of nitrogen base beads will be inaccessible to the inflowing probe c-ssDNA molecules since they are buried inside the matrix of condensed c-ssDNA polymer [Fig 2C].

### Calculation of the Nonspecific-Contact Formation Rate

Actually most of the theoretical models derived the scaling over the length of c-ssDNAs mainly from the fact that the radius of gyration (measured in bps) associated with reacting c-ssDNAs scales with their length as *r*_*M*_ ∝ *M*^*α*^ (*M* = *L* and *l* for the template and probe strands respectively). Here the value of the exponent 0 < *α* < 1 varies depending on the type of polymer and solvent conditions. For an ideal Gaussian chain polymer that is immersed in a theta solvent one finds that *α* ~ ½. For a linear chain polymer one finds that $r_{M}=\sqrt{Ml_{p}/6}$ (*M* = *L*, *l*) where *l*_*p*_ is the average bond length [32, 33]. For c-ssDNA we have *l*_*p*_ ~ 1 base between nitrogen base beads. Noting these facts the Smolochowski type limiting rate for a diffusion controlled nonspecific contact forming step can be given as follows.

$$k_{fQ}=B_{Q}J_{Q};\;J_{Q}=\left(D_{l}+D_{L}\right)/r_{Q};\;D_{M}=k_{B}T/6\pi \eta r_{M};\;Q=\left\{C,R\right\};\;M=\left\{L,l\right\}$$ (2)

Here *J*_*Q*_ is the inflowing flux of c-ssDNA molecules, *B*_*Q*_ is the exposed total surface area on which the influx of c-ssDNA molecules *J*_*Q*_ is acting, *D*_*S*_ is the three-dimensional (3D) diffusion coefficient associated with the colliding c-ssDNAs and *r*_*Q*_ is the reaction radius that depends on the conformational state of c-ssDNA. When c-ssDNA strands are relaxed, then all the base beads will be distributed over the surface of the solvent shell that covers the entire c-ssDNA polymer. Since *V*_*M*_ > *C*_*M*_ for *M* = {*L*, *l*} there will be several patches on the solvent shell surface without base beads. Noting that a nonspecific contact can be formed only upon collision between probe and base beads of template, one needs to integrate over the surface of the template c-ssDNA polymer that is spread over the surface of spherical solvent shell rather than the entire surface of spherical solvent shell [Fig 2B]. In this situation one finds that *B*_*R*_ ≃ 2*πr*_*R*_*L* where *r*_*R*_ = *r*_*D*_ + *r*_*l*_ is the reaction radius associated with the collision of the probe c-ssDNA strand on a nitrogen base bead of template c-ssDNA strand. On the other hand, when the c-ssDNA polymer is highly condensed then significant fraction of the nitrogen base beads will be buried inside the matrix of the DNA condensate [Fig 2C].

Unlike the relaxed conformational state, in case of condensed conformational state the surface of the spherical solvent shell that covers entire c-ssDNA strands will be filled with nitrogen base beads. This means that collision between base bead clusters of c-ssDNA strands always result in the formation of nonspecific contacts under condensed conformational state. In this condition one finds that $B_{C}≃4\pi r_{C}^{2}$. Here *r*_*C*_ = *r*_*L*_ + *r*_*l*_ is the reaction radius associated with the collision between the condensed nitrogen base bead clusters of template and probe c-ssDNA strands. Therefore depending on the conformational state of the colliding c-ssDNA molecules the bimolecular collision rate associated with the formation of nonspecific contact between the template and probe molecules can be written as follows.

Case I: Relaxed conformational state of c-ssDNA

$$k_{fR}≃2\pi r_{R}LJ_{R};\;J_{R}≃\left(D_{l}+D_{L}\right)/r_{R};\;r_{R}=r_{D}+r_{l};\;∴k_{fR}≃k_{t}L\left(1/r_{l}+1/r_{L}\right)/8$$ (3)

Case II: Condensed conformational state of c-ssDNA

$$k_{fC}≃4\pi r_{C}^{2}J_{C};\;J_{C}≃\left(D_{l}+D_{L}\right)/r_{C};\;r_{C}=r_{L}+r_{l};\;∴k_{fC}≃k_{t}{\left(r_{L}+r_{l}\right)}^{2}/4r_{L}r_{l}$$ (4)

Here *k*_*t*_ ≃ (8*k*_*B*_*T*/3*η*) is the Smolochowski type 3D diffusion controlled collision rate limit (M^-1^s^-1^) associated with the bimolecular collisions between the c-ssDNA molecules. Here *k*_*B*_ is the Boltzmann constant, *η* is the viscosity of the reaction medium and *T* is the absolute temperature in degrees *K*. Further the colliding molecules are assumed to be same in size with no charge on them. In general *k*_*fQ*_ ≃ *k*_*t*_*δ*_*Q*_ where *δ*_*R*_ ≃ *L*(1/*r*_*l*_ + 1/*r*_*L*_)/8 and *δ*_*c*_ ≃ (*r*_*L*_ + *r*_*l*_)^2^/4*r*_*L*_*r*_*l*_. When the colliding c-ssDNA strands are same in size then *δ*_*R*_ ≃ *L*/4*r*_*L*_ and *δ*_*c*_ ≃ 1. Noting that [32–33] for a linear chain polymer $r_{L}=\sqrt{Ll_{p}/6}$ one obtains $\delta_{R}≃\sqrt{3L/8l_{p}}$.

### Role of Electrostatic Repulsions at the DNA-DNA Interface

While deriving Eqs 3 and 4 we have not considered the electrostatic repulsions between the negatively charged phosphate backbones of c-ssDNA chains and shielding effects of solvent and other ion molecules present at the DNA-DNA interface of c-ssDNA molecules. Upon considering this fact and following the detailed works of Montroll in Ref. [34] we find the expression for the modified bimolecular rate constants as follows.

$$k_{fQ}=k_{t}\psi_{Q}\delta_{Q};\;\psi_{Q}≃\left(\kappa /r_{Q}\right)/\left(e^{\kappa /r_{Q}}-1\right);\;\kappa =\zeta_{S}\zeta_{S\prime }e^{2}/\mu k_{B}T;\;Q=\left(C,R\right)$$ (5)

Here *κ* is the Onsager radius which is defined as the distance between negatively charged phosphate backbones of colliding c-ssDNA chains at which the electrostatic repulsive energy is same as that of the thermal energy (~*k*_*B*_*T*), *ζ*_*S*_*e* and *ζ*_*S’*_*e* are the overall charges on the respective c-ssDNA molecules. Since |*κ*| ≥ *r*_*Q*_ by definition and *κ* > 0 in the present context, one finds that *ψ*_*Q*_ ~ |*κ*|/*r*_*Q*_ for large values of |*κ*| and for |*κ*| = *r*_*Q*_ one obtains *ψ*_*Q*_ ≃ 0.58. For a typical value of |*κ*| ≃ 10*r*_*Q*_ we find that *ψ*_*Q*_ ≃ 10^−4^. When *κ* < 0 as in case of site-specific DNA-protein interactions we obtain ${\text{lim}}_{\left|\kappa \right|\to r_{Q}}\psi_{Q}≃1.58$. One should also note that *ψ*_*Q*_ ≃ 1 only when we have |*κ*| = 0. While deriving Eq 5 we have not considered the shielding effects of solvent ions present at the DNA-DNA interface over the electrostatic repulsive forces between the phosphate backbones of c-ssDNA strands. Upon following the Debye theory of kinetic salt effects over diffusion controlled collision rate processes [35], one can rewrite the modified bimolecular rate constant in the presence of other ions in the solvent as follows.

$$k_{fQ}≃k_{t}\chi_{Q}\delta_{Q};\;\chi_{Q}=\psi_{Q}\text{exp}\left(2F\zeta_{S}\zeta_{S\prime }\sqrt{\Xi }\right);\;F≃0.509$$ (6)

Here Ξ is the overall ionic strength of the aqueous medium and *Q* = (*C*, *R*) as defined in Eqs 3–5 depending on the type of conformational state of colliding c-ssDNA polymers.

### Calculation of the Nucleation Time and Nucleation Rate

We learnt from recent computational studies [29] that the colliding c-ssDNA with nonspecific contacts between them undergo several trials of slithering and internal displacement dynamics before reaching the correct-contact and then nucleate the zipping process. These dynamical processes are similar to that of the facilitating 1D diffusional dynamics as in case of site specific DNA-protein interactions. Unlike the overall electrostatic attractive forces acting at the DNA-protein interface, in case of DNA renaturation there is a strong electrostatic repulsive force acting at the interface of cn-ssDNA that will be shielded by the solvent molecules present at the interface of cn-ssDNA strands. With this background one can model the slithering dynamics as 1D diffusion of one c-ssDNA molecule on the other in the process of searching for the correct-contact. To find the correct-contact on one c-ssDNA the other c-ssDNA needs to try out at least *λ* = *L*/*n* stretches of 1D slithering with an average size of *n* bases. This will ensure that the initial nonspecific contact visits all the possible positions and subsequently the correct contact is formed. The mean first passage time (*τ*_*N*_) associated with the visit of all the possible positions of c-ssDNAs by the initial nonspecific-contact between them via 1D diffusion can be given as $\tau_{N}=\lambda {\bar{\tau }}_{c}$ where ${\bar{\tau }}_{c}≃n^{2}/12D_{o}$ is the average time that is required by an unbiased 1D random walker to visit *n* sites of a linear lattice [36–38] confined inside the interval *x* ∈(0, *n*) starting from anywhere inside the interval as shown in **Appendix A**.

Noting that the nucleation rate is given as *k*_*N*_ = 1/*τ*_*N*_ one can obtain *k*_*N*_ ≃ 12*D*_*o*_/*nL* which means that *k*_*N*_ ∝ 1/*L*. This is a reasonable one since the probability of finding the correct contact upon each 3D diffusion mediated nonspecific collisions between c-ssDNA strands is *p*_*cc*_ = 1/*L*. One should also note that the probability of finding the correct contact upon each nonspecific collisions will be independent on the repetitiveness of c-ssDNA strands. Here *D*_*o*_ is the one dimensional diffusion coefficient associated with the dynamics of the probe c-ssDNA strand over the template c-ssDNA strand. One should note that *D*_*o*_ includes the contributions from both slithering and internal displacement dynamics which occurs within a cn-ssDNA molecule. Actually one can write down the expression for the 1D diffusion coefficient as follows.

$$D_{o}=D_{os}+D_{oi};\;D_{os}≃l_{p}^{2}\left(p_{f}w_{f}+p_{r}w_{r}\right);\;D_{oi}≃l_{d}^{2}\left(p_{f,d}w_{f,d}+p_{r,d}w_{r,d}\right)$$ (7)

Here *D*_*os*_ is the diffusion coefficient corresponds to slithering dynamics and *D*_*oi*_ corresponds to the internal displacement dynamics where *p*_*f/r*_ are the transition probabilities associated with the forward and reverse steps of the random walker with step size *l*_*p*_ = 1 base and *w*_*f/r*_ are the corresponding transition rates. Similarly in case of internal displacement dynamics *l*_*d*_ is the average step size and *p*_*f/r*,*d*_ are the respective forward and reverse transition probabilities and *w*_*f/r*,*d*_ are the corresponding transition rates. Here we consider the average step size since the step size associated with the inchworm type movements in the internal displacement mechanism is a random variable. For an unbiased searching via both 1D slithering and internal displacements we have *p*_*f/r*_ = *p*_*f/r*,*d*_ = 1/2.

Although *l*_*d*_ > *l*_*p*_, internal displacement dynamics may not be able to contribute much to the overall searching dynamics under relaxed conformational state of the colliding c-ssDNA strands since it requires looping and segmental motion of c-ssDNA chains. Therefore in general we have *w*_*f/r*_ > *w*_*f/r*,*d*_. Further one should note that slithering dynamics is a purely a local one i.e. slithering involves the local movement of bases. This means that the transition rates *w*_*f/r*_ and diffusion coefficient *D*_*os*_ will be independent of the length of c-ssDNA. Whereas internal displacement involves the movement of bulky segments [29] of the colliding c-ssDNA strands and therefore the corresponding transition rates *w*_*f/r*,*d*_ and diffusion coefficient *D*_*oi*_ will be dependent on the size of c-ssDNA strands. Here we can ignore the contribution of *D*_*oi*_ to the overall diffusion coefficient *D*_*o*_ only for the relaxed conformational state of c-ssDNA and we cannot ignore it for the condensed conformational state of c-ssDNA since the contribution from internal displacement mechanism will be the dominating one under such conditions.

### Calculation of the Zipping Time

Formation of correct-contact will result in the nucleation of zipping process. Upon formation of a nucleation site, the subsequent stochastic zipping of cc-ssDNA can be described by the following birth-death master equation.

$$\partial_{t}P\left(u,t\right)=k_{+}P\left(u-1,t\right)+k_{-}P\left(u+1,t\right)-\left(k_{+}+k_{-}\right)P\left(u,t\right)$$ (8)

Here *P*(*u*,*t*) = *P*(*u*,*t*|*u*_0_,*t*_0_) is the probability of finding the cc-ssDNA with *u* numbers of correct contacts at time *t* starting from the nucleation at *t* = *t*_*0*_ with *u* = *u*_*0*_ numbers of correct contacts, *k*_*+*_ (s^-1^) and *k*_*-*_ (s^-1^) are the respective average forward and reverse rate constants associated with the microscopic zipping reaction. Here the initial and boundary conditions corresponding to Eq 8 can be written as follows.

$$P\left(u,t_{0}\right)=P\left(u,t_{0}|u_{0},t_{0}\right)=\delta \left(u-u_{0}\right);\;k_{-}P\left(1,t\right)=k_{+}P\left(0,t\right);\;P\left(\beta +1,t\right)=0$$ (9)

Here *u* = 1 is a reflecting boundary and *u* = *β* is the absorbing boundary. One can solve the difference equation Eq 8 as follows. By defining equilibrium constant as *K*_*Z*_ = (*k*_-_/*k*_+_), one can find the following expression for the overall mean first passage time associated with complete zipping of *β* correct contacts (*u* = *β*) of cc-ssDNA starting from the number of correct contacts *u* = 1 as shown in **Appendix B**.

$$\tau_{Z}={\sum_{u=1}^{\beta }\phi \left(u\right)\sum_{w=1}^{u}\left(k_{+}\phi \left(w\right)\right)}^{-1}=\left(K_{Z}^{\beta +1}-K_{Z}\left(\beta +1\right)+\beta \right)/k_{+}{\left(1-K_{Z}\right)}^{2}$$ (10)

From this equation we find the limits ${\text{lim}}_{K_{Z}\to 1}\tau_{Z}≃\beta \left(1+\beta \right)/2k_{+}$ and ${\text{lim}}_{K_{Z}\to 0}\tau_{Z}≃\beta /k_{+}$ where *β* = *L/l*_*p*_ is a dimensionless quantity which is the total number of correct-contacts between colliding c-ssDNA upon formation of dsDNA. Here *L* is total length of c-ssDNA in bases and *l*_*p*_ = 1 base (1 base ~ 3.4x10^-10^ m). When the forward rate constant associated with formation of dsDNA is much higher than the reverse rate constant then we find the expression for the zipping rate constant as that *k*_*Z*_ = 1/*τ*_*Z*_ ≃ *k*_*p*_/*L* where we have defined *k*_*p*_ = *k*_*+*_*l*_*p*_ (bases/s). On the other hand when *k*_*+*_ = *k*_*-*_ then the zipping will be a pure 1D diffusion process with the phenomenological diffusion coefficient as $D_{\pm }≃l_{p}^{2}k_{+}$(base^2^/s) and subsequently *τ*_*Z*_ ≃ *L*^2^/2*D*_±_. When the reacting c-ssDNA is repetitive with a sequence complexity of *c* bases (here we have *c*∈(1, *L*)), then the observed zipping rate will be proportional to the number of repeats in that template c-ssDNA strand (*ρ* = *L*/*c*) and one obtains ${\text{lim}}_{K_{Z}\to 0}\tau_{Z}≃c/k_{p}$ i.e. the total zipping time will be directly proportional to the complexity of the reacting c-ssDNA molecules which is in line with the experimental observations [6–7]. Using these results one can write down the expression for the total time that is required for the overall nucleation and zipping processes (*τ*_*NZ*_) to generate dsDNA as follows.

$$\tau_{NZ}=\tau_{N}+\tau_{Z};\;{\text{lim}}_{K_{Z}\to 0}\tau_{Z}≃c/k_{p};\;{\text{lim}}_{K_{Z}\to 1}\tau_{Z}≃Lc/2D_{\pm };\;\tau_{N}=\lambda n^{2}/12D_{o}\text{; }\lambda =L/n$$ (11)

Here one should note that the zipping process can be thought as a random walk over a linear lattice [36–38] with initial and boundary conditions given in Eq 9. The phenomenological diffusion coefficient associated with such random walk is defined as $D_{\pm }≃l_{p}^{2}\left(p_{+}k_{+}+p_{-}k_{-}\right)$ where *p*_±_ are the transition probabilities associated with the forward and reverse steps in the zipping process. When the zipping is unbiased over forward or reverse steps with *k*_*+*_ = *k*_*-*_ then *p*_±_ = 1/2 and we recover the expression for $D_{\pm }≃l_{p}^{2}k_{+}$ in the limit as *K*_*Z*_ = 1.

### Calculation of the Overall Renaturation Rate

Using the expressions for the nucleation and zipping times one can define the overall bimolecular collision rate associated with the complete formation of dsDNA from c-ssDNA in **Scheme III** of Fig 2 as follows.

$${\bar{k}}_{HQ}=\int_{0}^{L}k_{HQ}p\left(n\right)dn;\;k_{HQ}=k_{fQ}/\left(1+k_{r}\tau_{NZ}\right)≃k_{fQ}/\left(1+nL/Y_{A}^{2}+c/Y_{E}\right)$$ (12)

**Fig 4:**
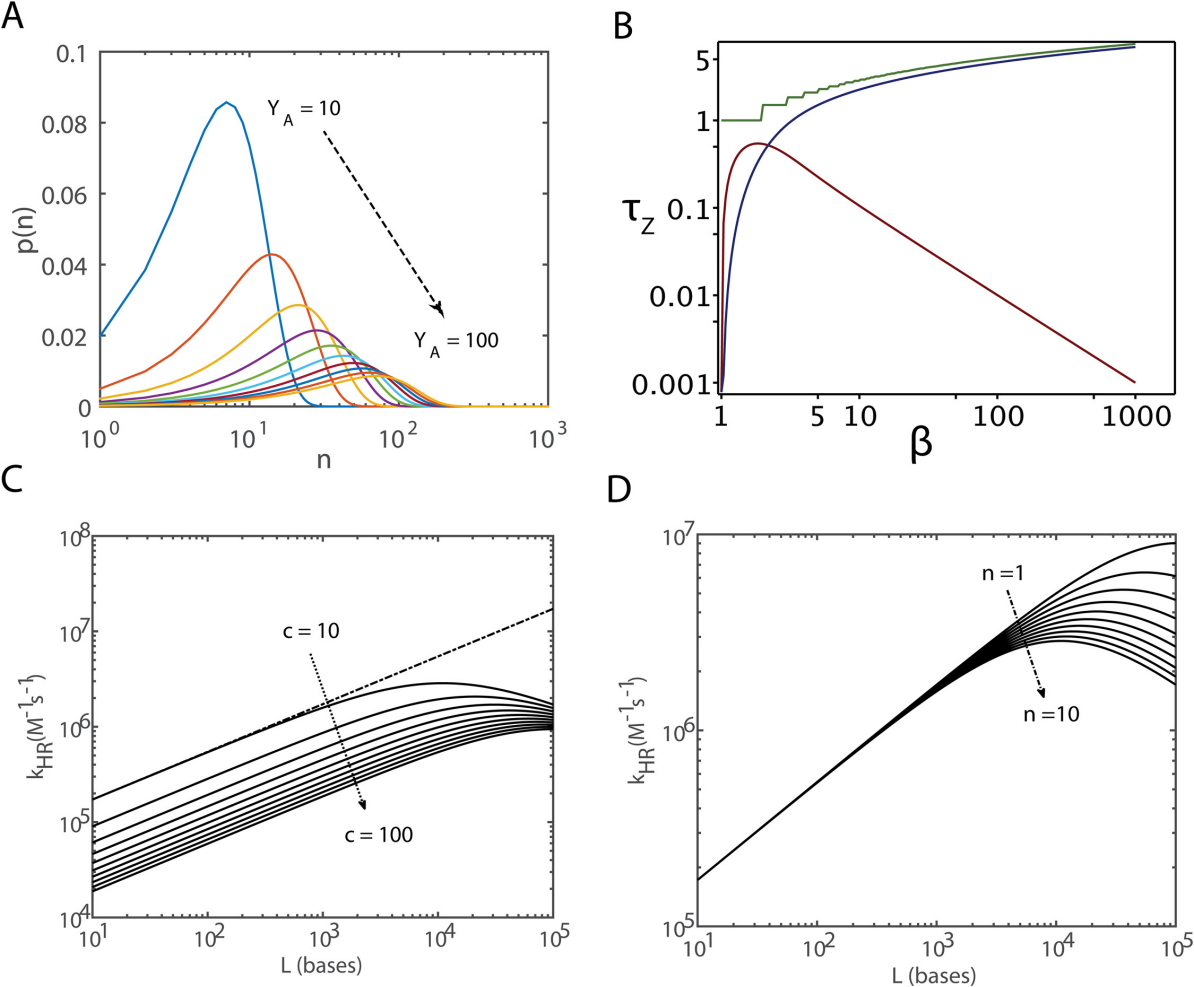
Cooperative effects on DNA renaturation. **A**. Probability density function associated with the one dimensional slithering length (*n* measured in bases) of cn-ssDNA in the process of searching for the correct-contact as given in Eq 13 for different values of the characteristic length $Y_{A}=\sqrt{12D_{o}/k_{r}}$ ranging from 10 to 100 bases where *D*_*o*_ (base^2^s^-1^) is the one dimensional diffusion coefficient associated with the slithering dynamics and *k*_*r*_ is the dissociation rate constant connected with cn-ssDNA. **B**. Zipping time (*τ*_*Z*_, measured in seconds) in the presence of cooperative effects. Here sequence complexity (*c*) is same as that of the length (*L*) of c-ssDNA i.e. *c* = *L*. The number of correct-contacts *β* = *L*/*l*_*p*_ is a dimensionless quantity where *l*_*p*_ = 1 base and *L* is the length of the reacting c-ssDNA. Green solid line is calculation from Eq 18 and blue solid line is calculation from Eq C9 of **Appendix C**. Here we have set *K*_*Z*_ ~ 10^−6^ and *k*_*+*_ ~ 1 s^-1^. Red solid line is the derivative of zipping time with respect to *β* as in Eq 21 which shows that the value of the derivative of overall zipping time with respect to *β* is < 10^−2^ when *β* > 10^2^. These plots suggest that when *K*_*Z*_ tends towards zero, the overall zipping time of a non-repetitive and long c-ssDNA will be independent of the length of the reacting c-ssDNA molecules. Zipping time of a repetitive c-ssDNA with a sequence complexity of *c* bases increases linearly with *c*. **C**. Variation of the overall renaturation rate *k*_*HR*_ with respect to length and complexity of c-ssDNA under relaxed conformational state. Here settings are $k_{fR}≃6\times 10^{5}\sqrt{L}$ M^-1^s^-1^, *Y*_*A*_ = 100 bases, *Y*_*E*_ = 1 bases and *n* = 10 bases. **D**. Variation of the overall renaturation rate *k*_*HR*_ as in Eq 12 with respect to the length of reacting c-ssDNA strands *L* and 1D slithering distance *n* under relaxed conformational state. Here settings are $k_{fR}≃6\times 10^{5}\sqrt{L}$ M^-1^s^-1^, *Y*_*A*_ = 100 bases, *Y*_*E*_ = 1 bases and *c* = 10 bases. In both **C** and **D**, *k*_*HR*_ shows a maximum at *L* = *L*_*opt*_. Here Lopt can be obtained by solving *∂*_L_*k*_*HR*_ = 0 for *L*. Explicitly one finds that $L_{opt}≃Y_{A}^{2}\left(c+Y_{E}\right)/nY_{E}$. The dotted line in (**C)** is $k_{HR}≃6\times 10^{5}\sqrt{L}/\left(1+c/Y_{E}\right)$ which breaks down beyond *L*_*opt*_.

Here we have defined two important characteristic lengths $Y_{A}=\sqrt{12D_{o}/k_{r}}$ and *Y*_*E*_ = *k*_*p*_/*k*_*r*_. The length *Y*_*A*_ describes the distance that is travelled by the initial nonspecific contact before cn-ssDNA dissociates whereas *Y*_*E*_ describes the distance travelled in the zipping reaction before cn-ssDNA dissociates into corresponding c-ssDNA molecules. Generally one observes that *Y*_*A*_ > *Y*_*E*_. The probability density function connected with the 1D slithering lengths *n* or its weighting function *p*(*n*) can be calculated as follows. When the residence times (*τ*) associated with the dissociation of cn-ssDNA molecules is distributed as an exponential then one finds that $p\left(\tau \right)\propto e^{-k_{r}\tau }$ and subsequently one obtains $p\left(n\right)\propto ne^{-{\left(n/Y_{A}\right)}^{2}}$ which mainly originates from the fact that within the residence time *τ*, the distance travelled by the nonspecific contact through 1D diffusion dynamics can be anywhere in the interval *n*∈(1, *L*) so that we obtain the transformation rule as *τ = n*^2^/12*D*_*o*_. With this definition of the residence time of c-ssDNA strands in the cn-ssDNA configuration one can write down the expression for the distribution of slithering lengths *n* as follows [Fig 4A].

$$p\left(n\right)=2ne^{-{\left(n/Y_{A}\right)}^{2}}/Y_{A}^{2}\left(1-e^{-{\left(L/Y_{A}\right)}^{2}}\right)≃2ne^{-{\left(n/Y_{A}\right)}^{2}}/Y_{A}^{2}$$ (13)

Using the expression of *p*(*n*) in Eq 13 and expanding *k*_*HQ*_ in a Maclaurin series one can obtain the following expression for the overall renaturation rate constant.

$${\bar{k}}_{HQ}≃\left(k_{fQ}/\left(1+c/Y_{E}\right)\right)\sum_{m=0}^{\infty }\Gamma \left(m/2+1\right){\left(-L/Y_{A}\left(1+c/Y_{E}\right)\right)}^{m}$$ (14)

While deriving this equation without losing generality we have extended the limits of *n* in the integration towards infinity since both *p*(*n*) and *k*_*HQ*_ approach zero at this limit. Under certain conditions one can obtain the following approximation for the series in Eq 14.

$${\bar{k}}_{HQ}≃\left(k_{fQ}Y_{E}/c\right)\sum_{m=0}^{\infty }\Gamma \left(m/2+1\right){\left(-\rho Y_{E}/Y_{A}\right)}^{m};\;Y_{A}\gg Y_{E};\;\left(c/Y_{E}\right)\gg 1$$ (15)

Later we will show that these inequality conditions are indeed valid. When the sequence complexity of the template c-ssDNA molecule is high enough and (*pY*_*E*_/*Y*_*A*_) ≪ 1 then one can write down the leading zeroth order approximation (*m* = 0 in Eqs 14 and 15) of the overall second order rate constant associated with the renaturation of relaxed c-ssDNA chains with equal lengths *L* = *l* as follows.

$${\bar{k}}_{HR}≃k_{fR}Y_{E}/c=k_{fR}k_{p}/k_{r}c=k_{t}\chi_{R}k_{p}L/4r_{L}k_{r}c\propto \sqrt{L}/c\eta ;\;∵r_{L}\propto \sqrt{L};\;k_{t}\propto 1/\eta$$ (16)

Upon substituting the expression $r_{L}≃\sqrt{Ll_{p}/6}$ in Eq 16 one obtains that ${\bar{k}}_{HR}≃k_{sm}\sqrt{Ll_{p}}/c$ in line with the expression for the overall renaturation rate in Wetmur-Davidson model [6]. Here *k*_*sm*_≃*k*_*t*_*ε* is the Smolochowski type 3D diffusion controlled collision rate limit corresponding to a situation *L* = *c* = 1 where we have defined $\varepsilon ≃\chi_{R}\left(k_{+}/k_{r}\right)\sqrt{3/8}$. When the conditions given in Eq 15 are true then Eq 16 suggests that the overall bimolecular collision rate associated with the renaturation reaction is directly proportional to the square-root of the length of c-ssDNA and inversely proportional to both sequence complexity of the reacting c-ssDNA molecules and viscosity of the reaction medium in line with the experimental observations. Experiments suggest that the scaling of renaturation rate with the length of c-ssDNA molecules that is given in Eq 16 is valid [6] only in the range of *L* ~ 10^2^−10^4^ bases. In this context our model suggest that the square root scaling of the renaturation rate on length of reacting c-ssDNA strands will be valid only when the inequalities given in Eq 15 are true apart from the condition that (*pY*_*E*_/*Y*_*A*_) ≪ 1 which may break down beyond certain values of the copy number (*ρ*) in the repetitive c-ssDNA.

### Role of Cooperativity in Renaturation Kinetics

Scaling results given by Eq 16 works only for highly repetitive and relaxed conformational state of c-ssDNA (*L* > *c*) and it will break down at *L* = *c* since at this point the scaling becomes as ${\bar{k}}_{HR}\propto 1/\sqrt{L}$ which is not true. The main reason for this observation is that while calculating the zipping rate constant we have not considered the underlying cooperative effects over the zipping process. When c-ssDNA is highly repetitive then the zipping process can take place in parallel for all the *ρ* = (*L*/*c*) number of short repetitive motifs. Under such conditions the cooperative effects will not be noticeable since the enhancement of renaturation process by the parallel-zipping will dominate over the underlying cooperative effects. This means that Eq 16 will be true only for repetitive c-ssDNA.

When the reacting c-ssDNA molecules are non-repetitive and long enough then the probability of formation of an additional correct-contact in the cc-ssDNA molecule that is undergoing zipping reaction will be directly proportional to the existing number of correct-contacts (*u*) and the probability of breakdown of an existing correct-contact will be directly proportional to the number of overhanging single stranded stretches of cc-ssDNA (*β*-*u*). This is true since the existing correct-contacts always stabilize newly formed correct-contacts and overhang single stranded regions of cc-ssDNA always try to destabilize the newly formed correct-contacts. Here one should note that we are dealing with the cooperative effects at a mesoscopic level within an independent and single renaturing cc-ssDNA molecule rather than at macroscopic level where the descriptive parameter of the renaturation process will be the number of mol-bases in c-ssDNA (*n*_*SS*_) or dsDNA (*n*_*DS*_) form rather than the number of correct-contacts in cc-ssDNA (*u*) as in the current context.

At macroscopic level the rate of change in the number of mol-bases in ssDNA form in the process of zipping will be directly proportional to the number of mol-bases in ssDNA form as well as number of mol-bases in dsDNA form which results in a cooperative sigmoidal type time evolution of the renaturation process [16–17, 28] where the macroscopic kinetic rate equation will be written as *dn*_*DS*_/*dt* ∝ *n*_*cc*_(*n*_0_−*n*_*DS*_)*n*_*DS*_. Here *n*_*0*_ is the initial concentration of mol-bases of c-ssDNA molecules in the system and *n*_*cc*_ is the total number of cc-ssDNA molecules in the system. With this background the birth-death master Eq 8 can be rewritten to include the cooperative effects for renaturation of a single cc-ssDNA molecule as follows.

$$\partial_{t}P\left(u,t\right)=k_{+}\left(u-1\right)P\left(u-1,t\right)+k_{-}\left(\beta -u-1\right)P\left(u+1,t\right)-\left(k_{+}u+k_{-}\left(\beta -u\right)\right)P\left(u,t\right)$$ (17)

Upon solving the backward type equation corresponding to this differential difference equation for appropriate boundary conditions as shown in Eq 9 and **Appendix C** one obtains the following expression for the overall zipping time that is required for the formation of *u* = *β* numbers of correct-contacts starting from *u* = 1 in the presence of cooperative effects.

τZ=∑u=1βKZu(ξF21([1,1],[2−β],−KZ−1)+ϕF21([1,u+1],[u+2−β],−KZ−1)) (18)

Here _2_*F*_1_ is the hypergeometric function and we have defined various parameters as follows.

$$\xi ={\left(-1\right)}^{u}\Gamma \left(u+1-\beta \right)/k_{-}\Gamma \left(u+1\right)\Gamma \left(1-\beta \right)\left(\beta -1\right);\;\phi =\Gamma \left(u+1-\beta \right)/K_{Z}^{u+1}k_{+}\Gamma \left(u+2-\beta \right)$$ (19)

The hypergeometric function of type _2_*F*_1_ is defined as follows.

F21([a,b],g,w)=∑m=0∞wm(a)m(b)m/m!(g)m; (h)q=Γ(h+q)/Γ(h) (20)

One can simplify the complicated expression given by Eq 18 using Fokker-Planck equation especially for large values of *β* as shown in **Appendix C**. Upon defining ${\text{lim}}_{K_{Z}\to 0}\tau_{Z}={\tilde{\tau }}_{Z}$ one can derive the following approximate expression for the overall zipping time.

$${\tilde{\tau }}_{Z}=\left(2/k_{+}\right)\int_{1}^{\beta }e^{-2y}\left(\text{Ei}\left(1,-2\right)-\text{Ei}\left(1,-2y\right)\right)dy;\text{ Ei}\left(a,z\right)=\int_{1}^{\infty }e^{-mz}m^{-a}dm$$ (21)

It seems from Eq 21 along with other computational analysis that the dependency of overall zipping time decreases with increasing *β* in the presence of cooperative effects which can be demonstrated by the following limiting conditions.

$$\partial {\tilde{\tau }}_{Z}/\partial \beta ≃2e^{-2\beta }\left(\text{Ei}\left(1,-2\right)-\text{Ei}\left(1,-2\beta \right)\right)/k_{+};\;{\text{lim}}_{\beta \to \infty }\partial {\tilde{\tau }}_{Z}/\partial \beta =0$$ (22)

From Eqs 17–22 one finds that when *K*_*Z*_ tends towards zero then in the presence of strong cooperative effects the zipping time of a non-repetitive cc-ssDNA will be independent of the length of the reacting c-ssDNA molecules especially for large values of *L* as shown in Fig 4B. Based on these observations we recover the observed scaling of the overall bimolecular rate constant on the length of the colliding c-ssDNA molecules as ${\bar{k}}_{HR}\propto \sqrt{L}/\eta$ for the renaturation of the non-repetitive c-ssDNA strands for which *L* = *c* in Eqs 15 and 16 since *Y*_*E*_ will be independent of sequence length and the number of copies will be *ρ* = 1.

## Discussion

### Two-Step DNA Renaturation Model of Wetmur and Davidson

Understanding the mechanism of renaturation of c-ssDNA is one of the central topics in molecular biology and biological physics. Wetmur and Davidson [6] developed their model by assuming that the renaturation rate is directly proportional to the total phosphate concentration which is in turn directly proportional to the total number of mol-bases in ssDNA or dsDNA form. According to their model the overall second order rate (*k*_*2*_) associated with the renaturation of repetitive c-ssDNA can be written as *k*_2_∝*k*_*N*_*p* where *ρ* = *L*/*c* is the copy number of repetitive motifs in the entire c-ssDNA polymer. Here *L* is the length of c-ssDNA and *c* is the sequence complexity and the nucleation rate was assumed to scale with *L* as *L*^*-α*^ where *α* = 1/2 due to the excluded volume effects associated with the interpenetration of c-ssDNA molecules that is essential for the nucleation reaction. As a result one obtains the scaling as $k_{2}\propto \sqrt{L}/c$ where the proportionality constant was assumed to be the Smolochowski type bimolecular collision rate limit (*k*_*sm*_) i.e.$k_{2}=k_{sm}\sqrt{Ll_{p}}/c$.

In Wetmur-Davidson model it was assumed that *k*_*sm*_ = *k*_*t*_. Here one can identify that $k_{2}={\bar{k}}_{HR}$ and *k*_*sm*_ = (*k*_*t*_ *ε*) of our model Eq 16 particularly for a relaxed conformational state of c-ssDNA that also includes the contributions from the electrostatic repulsions at the interface of colliding c-ssDNA molecules. Since in this model the nucleation is combined with nonspecific-contact formation step one finds that the nucleation rate is inversely proportional to the square root of the length of c-ssDNA strands. The main arguments for this scaling result put forth by Wetmur and Davidson were viz. (**a**) the radius of gyration of c-ssDNA is directly proportional to its length and (**b**) the reaction radius associated with the collision between c-ssDNA molecules is independent of the radius of gyration of c-ssDNA chains since both these strands can interpenetrate freely upon their collision. Though the assumption (**a**) is a right one for Gaussian chain polymers there are several questions associated with the assumption (**b**) since the reaction radius always depends on the sum of the radii of gyration of reactant molecules. In this context our detailed model clarifies the origin of such scaling in the renaturation phenomenon. It is clear from our theory that the rate constant associated with the formation of initial nonspecific-contact is directly proportional to the square-root of the length of the reacting c-ssDNA molecules.

One should note that it is very difficult to identify and isolate nucleated cc-ssDNA molecules since they are indistinguishable from the zipping cc-ssDNA molecules. Therefore it is more appropriate to combine the nucleation step with the zipping step rather than with the nonspecific-contact formation step as in case of Wetmur-Davidson model. Other issues in their model are such as the breakdown of scaling at *L* = *c* arises because the underlying cooperative effects in the long and non-repetitive c-ssDNA were not considered in their model as in case of our Eq 16. Further upon extrapolating towards the limit *L* = 1 base (so that the sequence complexity becomes as *c* = 1 base) Wetmur-Davidson model predicted that *k*_2_ = *k*_*t*_. However experimental observations suggested that the extrapolated bimolecular collision rate constant associated with the renaturation reaction at *L* = *c* = 1 was ~10^3^ times lower than the Smolochowski type diffusion controlled bimolecular collision rate limit (*k*_*t*_). On this basis they in turn discarded the possibility of diffusion control in the kinetics of renaturation of c-ssDNA molecules.

In this context Eq 16 of our model suggests an approximate expression for the extrapolation intercept as *k*_*sm*_ ≃ *k*_*t*_*ε* from which we can deduce that $\varepsilon ≃\chi_{R}\left(k_{+}/k_{r}\right)\sqrt{3/8}≃10^{-3}$. One should note that **scheme I** of Fig 1 is still valid with zero nucleation and zipping times and the steric factor *ε* mainly accounts for the geometric constraints associated with the bond formation between nitrogen bases A-T or G-C of the colliding single nucleotides in the limit *L* = 1 and *c* = 1 apart from the electrostatic repulsions due to the negatively charged phosphate groups. Here one should note that in our model the square-root dependency of renaturation rate on the length of c-ssDNA molecules mainly originates from the fact that the radius of gyration of c-ssDNA molecules scales with length as *r*_*L*_ ∝ *L*^α^ where *α* = ½ which is valid only for a Gaussian type polymers in a theta solvent. It seems that the error introduced by this assumption in the exponent is within the experimental error range [6].

### Temperature Dependency of DNA Renaturation Rate

Although the dissociation rate (here it is *k*_*r*_) constant increases exponentially with temperature [39–42] there are several controversies exist on the dependency of renaturation rate constant on increasing temperature. Some experimental studies established [39–40] a decrease in the renaturation rate constant with increase in temperature and some other studies have shown an increase in the renaturation rate constant with increase in temperature [8, 41]. In general it seems that the temperature dependency of the renaturation rate constant is of non-Arrhenius one and non-monotonic type [42]. Simulation studies suggested that there exists an optimum temperature at which the renaturation rate constant is a maximum [29]. In our model the nonspecific-contact formation, nucleation and zipping steps are all influenced by the rise in temperature in a complicated way.

Actually in Eq 16, the Smolochowski type bimolecular collision rate constant depends on temperature as *k*_*t*_ = (8*k*_*B*_*T*/3*η*) where we assume that viscosity of the medium is not changing much in the range of temperature variation and the dissociation rate scales with temperature as $k_{r}=k_{r}^{0}\text{exp}\left(-\omega /k_{B}T\right)$ in line with transition state theory where *ω* is the free energy barrier associated with the dissociation of cn-ssDNA complex. The rate constant associated with the microscopic zipping (*k*_*p*_ ~ *l*_*p*_*k*_*+*_) is connected with the microscopic diffusion coefficient *D*_±_ ~ *l*_*p*_^2^*k*_+_ associated with the zipping reaction. Apart from these the dimensionless parameter *χ*_*R*_ corresponding to the overall electrostatic repulsions and the shielding effects of solvent ions at the interface of cn-ssDNA molecules also depends on the temperature as given in Eqs 5 and 6. It seems that the non-Arrhenius type kinetic behaviour arises as a consequence of a complicated interplay between increase in the rate of nonspecific-contact formation and combined effects of increase in the dissociation rate constant and microscopic zipping rate constant as the temperature increases from low to high values. Since ${\bar{k}}_{HR}\propto k_{B}T\text{exp}\left(\omega /k_{B}T\right)$ one finds that $\text{ln}{\bar{k}}_{HR}$ will be a maximum approximately at *T* ~ *ω/k*_*B*_ as observed in the simulation studies [29].

### Comparison with Experimental Data on DNA Renaturation

Under relaxed conformational state, the 3D diffusion mediated nonspecific contact formation rate scales with the size of c-ssDNA strands as $k_{fR}≃k_{t}\chi_{R}\sqrt{3L/8l_{p}}$. Since the negatively charged phosphate backbones of c-ssDNA repel each other the Onsager radius (*κ*) associated with the collision of c-ssDNA strands will be much higher than the reaction radius (*r*_*R*_) under relaxed conformational state. When *κ* ~ 10*r*_*R*_ then one obtains *χ*_*R*_~10^−3^. Noting that *k*_*t*_~10^9^ M^-1^s^-1^ and *l*_*p*_ ~ 1base one finds that $k_{fR}≃6\times 10^{5}\sqrt{L}$ M^-1^s^-1^. In the calculation of *k*_*t*_ we have used *T* = 298K and viscosity coefficient *η* ~10^−1^ kgm^-1^s^-1^ for aqueous conditions. Single molecule experiments on DNA polymer in aqueous solution suggested [43–44] a 3D diffusion coefficient as $D_{L}≃D_{L}^{o}{\left(L/l_{p}\right)}^{-\alpha }$ where $D_{L}^{o}≃1.4\times 10^{10}$ base^2^s^-1^ and *α* ~ 0.59–0.71 depending on the condition of the aqueous medium. Furthermore the 3D diffusion coefficient seems to be several orders of magnitude less under crowded cytoplasmic environment [43]. Therefore the 1D diffusion coefficient associated with the slithering dynamics will be 10−10^2^ times slower than the 3D one since the local dynamics of individual bases which are involved in the slithering dynamics will be significantly restricted by the adjacent bases apart from the reduced degrees of freedom. In this background one can approximate the 1D diffusion coefficient as *D*_*o*_ ≃ 10^9^ base^2^s^-1^ under relaxed conformational state of c-ssDNA strands. Using this one can arrive at an empirical expression for the nucleation rate as *k*_*N*_ ≃ (10^9^/*nL*).

From experimental studies [41] one finds the zipping rate as *k*_*p*_ ~ 10^6^ bases/s which means that *k*_*+*_ ~ 10^6^ s^-1^. Using these values one obtains the zipping rate as *k*_*Z*_ ≃ (10^6^/*c*) s^-1^. Noting from experimental observations that $\varepsilon ≃\chi_{R}\left(k_{+}/k_{r}\right)\sqrt{3/8}$~10^−3^ and using the values of *k*_*+*_ and *χ*_*R*_ one can obtain the value of the dissociation rate as *k*_r_~10^6^ s^-1^. Using the numerical values of (*D*_*o*_, *k*_*p*_ and *k*_*r*_) one finds that *Y*_*A*_ ~ 10^2^ bases and *Y*_*E*_ ~ 1 bases. Computational studies on the renaturation of short fragments of c-ssDNA strands suggested [29] the most probable value for the 1D slithering length as *n* ~ (4–10) bases. For the purpose of calculations we use *n* = 10 bases. From Eq 12 one finds that the overall renaturation rate *k*_*HR*_ will be a maximum at *L* = *L*_*opt*_ where $L_{opt}≃Y_{A}^{2}\left(c+Y_{E}\right)/nY_{E}$(Fig 4C and 4D). This can be obtained by solving *∂*_*L*_*k*_*HR*_ = 0 for *L*. Here *k*_*HR*_ scales with *L* as $k_{HR}\propto \sqrt{L}$ whenever *L*<*L*_*opt*_ since the nonspecific contact formation step will be the dominating component under such conditions. When *L* > *L*_*opt*_ then the scaling with *L* becomes as $k_{HR}\propto 1/\sqrt{L}$ since the nucleation and zipping steps will be the bottlenecks under such conditions. Upon substituting *L* = *L*_*opt*_ into *k*_*HR*_ one can obtain the maximum achievable bimolecular renaturation rate as $\text{ max}\left(k_{HR}\right)≃k_{t}\chi_{R}Y_{A}\sqrt{3/32nl_{p}\left(1+c/Y_{E}\right)}$ for the renaturation of repetitive c-ssDNA strands. When the sequence complexity *c* is much higher than the characteristic length *Y*_*E*_ then one can deduce that $\text{ max}\left(k_{HR}\right)\propto Y_{A}\sqrt{Y_{E}/nc}$ and the maximum achievable renaturation rate seems to be $\text{ max}\left(k_{HR}\right)≃3\times 10^{7}/\sqrt{nc}$ M^-1^s^-1^. Upon substituting the numerical values of *Y*_*A*_ and *Y*_*E*_ into the expression for *L*_*opt*_ one obtains *L*_*opt*_ ~ 10^4^*c*/*n*. For a sequence of c-ssDNA strands with a complexity of *c* ~ *n* one finds that *L*_*opt*_ ~ 10^4^ bases. From Eq 12 we find that the square root scaling of the renaturation rate on *L* will be valid only up to ~10^4^ bases in line with the experimental observations [6, 27].

Upon substituting the numerical values of (*Y*_*A*_, *Y*_*E*_ and *k*_*fR*_) into the expression for the renaturation rate *k*_*HR*_ one finds that $k_{HR}≃6\times 10^{5}\sqrt{L}/\left(1+10^{-4}nL+c\right)$. Interestingly one should note that this functional form will behave as $k_{HR}≃6\times 10^{5}\sqrt{L}/\left(1+c\right)$ whenever $L<\left(Y_{A}^{2}c/nY_{E}\right)$. When *L* < *L*_*opt*_ and *c* > 1 then one obtains that $k_{HR}≃6\times 10^{5}\sqrt{L}/c$. Remarkably this expression for the renaturation rate *k*_*HR*_ is much close to the experimentally obtained fitting function of Wetmur-Davidson for the overall bimolecular renaturation rate as $k_{2}≃3.5\times 10^{5}\sqrt{L}/c$ for the experimentally measured range of *L* from 10^2^ to 10^4^ bases (Eq. 20 in Ref. [6**]**). It is still not clear [27] whether this scaling relationship will be valid beyond this range of *L* or not. In this context our theory based on **Scheme III** predicts that the square root scaling of the overall renaturation rate on the length of c-ssDNA will break down beyond *L*_*opt*_. When the inequality conditions given in Eq 15 are not true then one can also show that the average renaturation rate ${\bar{k}}_{HR}$ will be a maximum at $L={\bar{L}}_{opt}$ where ${\bar{L}}_{opt}$ is the solution of $\partial_{L}{\bar{k}}_{HR}=0$ for *L* as follows.

$$\partial_{L}{\bar{k}}_{HR}=\int_{0}^{L}\left(\left(n-2L{\left(n/Y_{A}\right)}^{2}\right)e^{-{\left(n/Y_{A}\right)}^{2}}/g_{n}\right)dn+2L^{2}e^{-{\left(L/Y_{A}\right)}^{2}}/g_{L}=0;\;g_{b}=\left(1+bL/Y_{A}^{2}+c/Y_{E}\right)$$

Here the subscript *b* in *g*_*b*_ can take (*n*, *L*). Detailed analysis suggests an approximation as ${\bar{L}}_{opt}≃Y_{A}^{2}\left(c+Y_{E}\right)/\bar{n}Y_{E}$ where $\bar{n}≃\int_{0}^{\infty }np\left(n\right)dn≃\sqrt{\pi }Y_{A}/2$. Clearly we find that ${\bar{L}}_{opt}<L_{opt}$. This is because the probability density function associated with the 1D slithering lengths as defined in Eq 13 will be valid only when the sequence complexity *c* is much higher than the maximum possible 1D slithering length *Y*_*A*_. When *c* < *Y*_*A*_ then the slithering dynamics associated with a nonspecific contact present in between the cn-ssDNA strands can progress only for *c* number of steps. Beyond this point either dissociation of cn-ssDNA strands or relocation of the nonspecific contact to some other position of cn-ssDNA is necessary. Therefore under such conditions one can replace the probability density with a delta function as *p*(*n*) ≃ *δ*(*n*−*c*). This means that we need to substitute as $\bar{n}\sim c$ in our calculations whenever *c* < *Y*_*A*_ and one obtains ${\bar{L}}_{opt}≃Y_{A}^{2}/Y_{E}\sim 10^{4}$ in the present context.

### Justifications for the Three-Step Model

In **Scheme III** the nucleation rate is inversely proportional to the length and zipping rate in inversely proportional to the complexity of the c-ssDNA strands. Therefore it is reasonable to cluster both nucleation and zipping steps together and assume that they are the rate limiting ones compared to the nonspecific contact formation step. Unlike the rate of nucleation and zipping the nonspecific contact formation rate increases with the length of c-ssDNA in a square root manner. We substantiate the three steps of renaturation by the following reasons viz. (**a**) the underlying microscopic processes are clearly dissimilar in cases of nonspecific contact formation, nucleation and zipping. Here nonspecific contact formation is a pure 3D diffusion mediated collision process. Whereas nucleation involves a combination of 1D and 3D diffusion. The zipping step is pure 1D diffusion like process which progresses from a stable nucleus, and (**b**) the scaling relationships associated with the corresponding rates on the length of reacting c-ssDNA and sequence complexity are different from each other. Clearly nonspecific contact formation, nucleation and zipping are all phenomenologically distinct processes which substantiate our three-step model. Since the nucleation and zipping and parts of a continuous process and a nucleated cc-ssDNA molecule is indistinguishable from the zipping one we have combined the nucleation step with the zipping step for the purpose of computing the rate associated with the overall nucleation-zipping. Here one should note that cn-ssDNA which is the product of nonspecific contact formation step is distinct from nucleated/zipping cc-ssDNA molecule.

### Comparison with Earlier Diffusion Based Models

Since 3D diffusion based models predict the scaling of overall renaturation rate on the length and sequence complexity of c-ssDNA strands as *k*_*HR*_ ∝ *L*/*c* one can rule out the possibility of **Scheme I**. Two step models as in **Scheme II** correctly predict the scaling of renaturation rate on the length and complexity of the reacting c-ssDNA strands. However models based on **Scheme II** such as the Wetmur-Davidson model [6] fail when *L* = *c* apart from their inability to explain the discrepancy in the intercept value of the bimolecular renaturation rate at *L* = *c* = 1. Models based on transition rate theory cannot explain the inverse viscosity dependence of the overall renaturation rate.

Sikorav et.al [27] suggested a Kramer’s type expression for the bimolecular nucleation rate constant where the scaling dependency of the overall bimolecular collision rate associated with renaturation on the length of c-ssDNA mainly originates from the entropic component of free energy barrier. However in this model the reaction coordinate and origin of free energy barrier associated with the nucleation and zipping are not clearly defined. Further the exact mechanism of formation of nucleation sites is not clearly explained. On the other hand as correctly pointed out by them, one cannot explain all the experimental observations related to the entire process of renaturation of c-ssDNA molecules with purely diffusion-controlled formalism or transition state theoretical framework. From our model we can conclude that the nonspecific contact formation step is a pure three dimensional diffusion controlled collision rate processes whereas both nucleation and zipping steps involve a sequence of several microscopic crossings of free-energy barriers as well as one dimensional diffusion type slithering dynamics on a linear lattice.

### Effects of Conformational State of DNA on the Renaturation Rate

Condensed conformational state of c-ssDNA polymers is one more cause for the breakdown of the scaling of renaturation rate on the length of c-ssDNA that is given in Eq 16. When the colliding c-ssDNA molecules are in condensed conformational state then the rate constant associated with the nonspecific-contact formation step will be independent of the length of c-ssDNA when *L* = *l*. Under such conditions the overall second order rate constant associated with the renaturation of repetitive c-ssDNA chains will be inversely proportional to the sequence complexity. Here one should note that while deducing these facts we have not considered the condensation of both c-ssDNA molecules together which is known to enhance the overall renaturation rate over several orders of magnitude as in case of renaturation in the phenol-water interface [45]. Under such co-condensation of both strands of c-ssDNA the rate of nonspecific-contact formation is very large and the dissociation rate will be very small and the rate limiting steps are the nucleation and zipping ones.

## Conclusions

Renaturation (or hybridization) of complementary single strands of DNA is an important phenomenon in molecular biology and biological physics. Understanding the kinetic mechanism of renaturation is very much useful to further understand the winding-unwinding dynamics of double stranded DNA under both *in vitro* and *in vivo* conditions. Here we have developed a stochastic dynamics based model on the DNA renaturation phenomenon to explain various scaling behaviours of renaturation rate. According to our model there are at least three steps in the renaturation process viz. nonspecific-contact formation, stochastic nucleation and zipping. Most of the earlier two-state models combined nucleation with nonspecific-contact formation step. We argue that it is considerably meaningful when we combine the nucleation with the zipping since nucleation is the initial step of zipping. Nonspecific-contact formation step is a pure three-dimensional diffusion controlled collision process. On the other hand nucleation involves several rounds of one-dimensional slithering dynamics of one single strand of DNA on the other complementary strand in the process of searching for the correct-contact and initiate nucleation. Upon nucleation, the stochastic zipping follows to generate a fully renatured double stranded DNA.

It seems that the square-root dependency of the overall renaturation rate constant on the length of reacting single strands originates mainly from the geometric constraints in the diffusion controlled nonspecific-contact formation step. On the other hand the inverse scaling of the renaturation rate with the sequence complexity originates from the stochastic zipping which involves several rounds of crossing of free-energy barrier at microscopic level. When the sequence of renaturing single strands of DNA is repetitive with less complexity then the cooperative effects will not be noticeable since the parallel zipping will be a dominating enhancement factor. However for DNA strand with high sequence complexity and length one needs to consider the cooperative effects both at microscopic and macroscopic levels to explain various scaling and kinetic behaviours of the overall renaturation rate.

## Appendix

### A. Nucleation via 1D Diffusion over Linear Lattice

The searching of the non-specifically bound cn-ssDNA strands for the correct-contacts on each other can be modelled as 1D random walk on a linear lattice. The stochastic differential equation associated with such an unbiased random walk on a linear lattice can be written as follows [36–38].

$$dx/dt=\sqrt{2D_{o}}\Gamma_{t};\;x\in \left(0,n\right);\;\langle \Gamma_{t}\rangle =0;\;\langle \Gamma_{t}\Gamma_{t\prime }\rangle =\delta \left(t-t\prime \right)$$ (A1)

Here *x* is the position of the random walker on the linear lattice, *D*_*o*_ is the one dimensional diffusion coefficient associated with the dynamics of the random walker, Γ_*t*_ is the Gaussian white noise with mean and variance as given in Eq A1. The probability density function associated with the dynamics of such random walker obeys the following forward Fokker-Planck equation (FPE) with initial condition.

$$\partial p\left(x,t|x_{0},t_{0}\right)/\partial t=D_{o}\partial^{2}p\left(x,t|x_{0},t_{0}\right)/\partial x^{2};\;p\left(x,t0|x_{0},t_{0}\right)=\delta \left(x-x_{0}\right)$$ (A2)

The boundary conditions associated with Eq A2 are *p*(0,*t*|*x*_0,_*t*_0_) = *p*(*n*,*t*|*x*_0,_*t*_0_) = 0. Here *p*(*x*,*t*|*x*_0,_0) is the probability of observing the random walker at position *x* at time *t* with the condition that the random walker was at position *x*_*0*_ at *t* = *t*_*0*_. Settings *t*_*0*_ = 0, the general solution to Eq A2 for the appropriate initial and boundary conditions can be obtained by the method of Eigen function expansion using biorthogonal set as follows [36–38].

$$p\left(x,t|x_{0},0\right)=\left(2/n\right)\sum_{k=0}^{\infty }\text{exp}\left(-k^{2}\pi^{2}D_{o}t/n^{2}\right)\text{sin}\left(k\pi x_{0}/n\right)\text{sin}\left(k\pi x/n\right)$$ (A3)

The mean first passage time (MFPT) associated with the escape of a random walker obeying Eq A3 from the interval *x* ∈(0, *n*) starting from an arbitrary lattice point *x* inside the interval obeys the following backward type Fokker-Planck equation.

$$D_{o}\left(d^{2}\tau_{x}/dx^{2}\right)=-1;\;\tau_{0}=\tau_{n}=0;\;\tau_{x}=\left(n^{2}-x^{2}\right)/2D_{o};\;{\bar{\tau }}_{c}=\left(1/n\right)\int_{0}^{n}\tau_{x}dx≃n^{2}/12D_{o}$$ (A4)

Since the random walker can enter initially anywhere in interval *x* ∈(0, *n*) of linear lattice with equal probabilities one needs to average the computed MFPT *τ*_*x*_ over all the values of initial positions *x*. As in Eq A4 we find the initial position averaged value as ${\bar{\tau }}_{c}≃n^{2}/12D_{o}$. This is approximately the time that is required by a random walker to visit all the sites of a linear lattice confined inside the interval *x* ∈(0, *n*) starting from anywhere inside the interval. This is evident from the following arguments. When we introduce reflecting boundaries at *x* = 0 as well as *x* = *n* then [*∂*_*x*_*p*(*x*,*t*|*x*_0,_0)]_*x* = 0_ = [*∂*_*x*_*p*(*n*,*t*|*x*_0,_0)]_*x* = *n*_ = 0 are the corresponding boundary conditions. The probability density function associated with the dynamics of such a random walker confined inside those reflecting boundaries can be given as follows.

$$p\left(x,t|x_{0},0\right)=1/n+\left(2/n\right)\sum_{k=1}^{\infty }\text{exp}\left(-k^{2}\pi^{2}D_{o}t/n^{2}\right)\text{cos}\left(k\pi x_{0}/n\right)\text{cos}\left(k\pi x/n\right)$$ (A5)

From this equation one can conclude that *p*(*x*,*t*|*x*_0,_0) ≃ 1/*n* whenever *t* ≥ (*n*^2^/*π*^2^*D*_o_) which is close to the initial position averaged mean first passage time ${\bar{\tau }}_{c}$. In other words in the presence of reflecting boundaries at both the ends of the linear lattice, the probability of observing the random walker anywhere within those boundaries will be equal when $t>{\bar{\tau }}_{c}$.

### B. Zipping of Repetitive c-ssDNA Sequences

The zipping of cc-ssDNA strands will also be a stochastic process which can be described by the following birth-death master equation.

$$\partial_{t}P\left(u,t\right)=k_{+}P\left(u-1,t\right)+k_{-}P\left(u+1,t\right)-\left(k_{+}+k_{-}\right)P\left(u,t\right)$$ (B1)

Here *P*(*u*, *t*) = *P*(*u*,*t*|*u*_*0*_,*t*_*0*_) is the probability of finding the cc-ssDNA with *u* numbers of correct contacts at time *t* starting from the nucleation at *t* = *t*_*0*_ with *u* = *u*_*0*_, *k*_*+*_ (s^-1^) and *k*_*-*_ (s^-1^) are the respective average forward and reverse rate constants associated with the microscopic zipping reaction. Here the initial and boundary conditions corresponding to Eq B1 can be written as follows.

$$P\left(u,t_{0}\right)=P\left(u,t_{0}|u_{0},t_{0}\right)=\delta \left(u-u_{0}\right);\;k_{-}P\left(1,t\right)=k_{+}P\left(0,t\right);\;P\left(\beta +1,t\right)=0$$ (B2)

The mean first passage time associated with the complete zipping of cc-ssDNA strands obeys the following backward type master equation with similar boundary conditions.

$$k_{+}U\left(u\right)-k_{-}U\left(u-1\right)=-1;\;U\left(u\right)=\tau \left(u+1\right)-\tau \left(u\right);\;\tau \left(\beta +1\right)=0;\;\tau \left(-1\right)=\tau \left(0\right)$$ (B3)

Here *u* = 1 is a reflecting boundary and *u* = *β* is the absorbing boundary. One can solve the difference equation Eq B2 as follows. By defining equilibrium constant as *K*_Z_ = (*k*_−_/*k*_+_), Eq B2 can be rewritten in the following form.

$$k_{+}u\phi \left(u\right)\left[\Omega \left(u\right)-\Omega \left(u-1\right)\right]=-1;\;\phi \left(u\right)=\prod_{w=2}^{u}K_{Z};\;\Omega \left(u\right)=U\left(u\right)/\phi \left(u\right)$$ (B4)

Upon solving [36–38] this difference equation for the boundary conditions given in Eq B3 we find the following expression for the overall mean first passage time associated with complete zipping of *β* correct contacts (*u* = *β*) of cc-ssDNA starting from the number of correct contacts *u* = 1.

$$\tau_{Z}={\sum_{u=1}^{\beta }\phi \left(u\right)\sum_{w=1}^{u}\left(k_{+}\phi \left(w\right)\right)}^{-1}=\left(K_{Z}^{\beta +1}-K_{Z}\left(\beta +1\right)+\beta \right)/k_{+}{\left(1-K_{Z}\right)}^{2}$$ (B5)

### C. Cooperative Effects on Non-Repetitive c-ssDNA Sequences

In the presence of cooperative effects the probability of formation of an additional correct contact in cc-ssDNA will be directly proportional to the already exiting number of correct contacts. Similarly the probability associated with the breaking of a correct contact will be directly proportional to the already exiting single stranded overhangs of cc-ssDNA. In the background the birth-death master equation described by Eq B1 can be rewritten to include the cooperative effects for the renaturation of a nonrepetitive single cc-ssDNA as follows.

$$\partial_{t}P\left(u,t\right)=k_{+}\left(u-1\right)P\left(u-1,t\right)+k_{-}\left(\beta -u-1\right)P\left(u+1,t\right)-\left(k_{+}u+k_{-}\left(\beta -u\right)\right)P\left(u,t\right)$$ (C1)

The mean first passage time *τ*(*u*) associated with evolution of the system from correct-contact *u* = 1 to complete dsDNA form with correct-contacts *u* = *β* can be written as follows where *U*(*u*) and other boundary conditions are defined as in Eq B3.

$$k_{+}uU\left(u\right)-k_{-}\left(\beta -u\right)U\left(u-1\right)=-1;\;k_{+}u\phi \left(u\right)\left[\Omega \left(u\right)-\Omega \left(u-1\right)\right]=-1$$ (C2)

Here Ω(*u*) = *U*(*u*)/*ϕ*(*u*) and the function *ϕ*(*u*) is defined as follows.

$$\phi \left(u\right)=\prod_{w=1}^{u}K_{Z}\left(\beta -w\right)/w={\left(-K_{Z}\right)}^{u}\Gamma \left(u+1-\beta \right)/\Gamma \left(u+1\right)\Gamma \left(1-\beta \right);\;K_{Z}=k_{-}/k_{+}$$ (C3)

Upon solving the difference equation Eq C2 for appropriate boundary conditions one obtains the following expression for the overall zipping time that is required for the formation of *u* = *β* numbers of correct-contacts starting from *u* = 1 in the presence of cooperative effects.

τZ=∑u=1βKZu(ξF21([1,1],[2−β],−KZ−1)+ϕF21([1,u+1],[u+2−β],−KZ−1)) (C4)

Here _2_F_1_ is the hypergeometric function and we have defined various parameters as follows.

$$\xi ={\left(-1\right)}^{u}\Gamma \left(u+1-\beta \right)/k_{-}\Gamma \left(u+1\right)\Gamma \left(1-\beta \right)\left(\beta -1\right);\;\phi =\Gamma \left(u+1-\beta \right)/K_{Z}^{u+1}k_{+}\Gamma \left(u+2-\beta \right)$$ (C5)

The hypergeometric function of type _2_F_1_ is defined as follows.

F21([a,b],g,z)=∑m=0∞zm(a)m(b)m/m!(g)m; (h)q=Γ(h+q)/Γ(h) (C6)

To simplify the complicated expression for *τ*_*Z*_ in Eq C3 particularly for sufficiently large values of *β* one can approximate Eq C3 by the following continuous type Fokker-Planck equation (FPE) [36–38].

$$\partial_{t}P\left(u,t\right)=-\partial_{u}\left(A\left(u\right)P\left(u,t\right)\right)+\partial_{u}^{2}\left(B\left(u\right)P\left(u,t\right)\right)/2$$ (C7)

Here the drift and diffusion coefficients can be written as follows.

$$A\left(u\right)=k_{+}u-k_{-}\left(\beta -u\right);\;B\left(u\right)=k_{+}u+k_{-}\left(\beta -u\right)$$ (C8)

Eqs C7 and C8 suggest that in the presence of cooperative effects the phenomenological diffusion coefficient associated with the zipping dynamics (*D*_±_) will be dependent on the number of correct-contacts. Using the backward type FPE corresponding to Eq C7 one can obtain the mean first passage time associated with the evolution of the system starting from *u* = 1 to *u* = *β* as follows.

$$\tau_{Z}≃\left(2/k_{+}\right)\int_{1}^{\beta }\left(Η\left(y\right)/\Phi \left(y\right)\right)dy\text{; }Η\left(y\right)=\int_{1}^{y}\left(\Phi \left(w\right)/\left(w+K_{Z}\left(\beta -w\right)\right)\right)dw$$ (C9)

In this equation various functions and parameters are defined as follows.

$$\Phi \left(q\right)=\text{exp}\left(2\int_{1}^{q}p\left(w\right)dw\right);\;p\left(w\right)=A\left(w\right)/B\left(w\right)=\left(w-K_{Z}\left(\beta -w\right)\right)/\left(w+K_{Z}\left(\beta -w\right)\right)$$ (C10)

Computational analysis of Eqs C7 and C9 suggests that in the limit as *K*_*Z*_ tends towards zero, the overall zipping time *τ*_*Z*_ approximately scales with *β* as 1-e^-2*β*^. Upon defining the limit as ${\text{lim}}_{K_{Z}\to 0}\tau_{Z}={\tilde{\tau }}_{Z}$ one can derive the following expression for the overall zipping time.

$${\tilde{\tau }}_{Z}=\left(2/k_{+}\right)\int_{1}^{\beta }e^{-2y}\left(\text{Ei}\left(1,-2\right)-\text{Ei}\left(1,-2y\right)\right)dy;\text{ Ei}\left(a,z\right)=\int_{1}^{\infty }e^{-mz}m^{-a}dm$$ (C11)

This equation suggests that for a sufficiently large value of *β*, in the presence of cooperative effects the overall zipping time will be almost independent of *β* since ${\text{lim}}_{\beta \to \infty }\partial_{\beta }{\tilde{\tau }}_{Z}=0$.
